# Lysate of *Parabacteroides distasonis* prevents severe forms of experimental autoimmune encephalomyelitis by modulating the priming of T cell response

**DOI:** 10.3389/fimmu.2024.1475126

**Published:** 2024-12-16

**Authors:** Zuzana Jiraskova Zakostelska, Michal Kraus, Stepan Coufal, Petra Prochazkova, Zaneta Slavickova, Tomas Thon, Tomas Hrncir, Jakub Kreisinger, Klara Kostovcikova, Pavlina Kleinova, Jana Lizrova Preiningerova, Miluse Pavelcova, Veronika Ticha, Ivana Kovarova, Eva Kubala Havrdova, Helena Tlaskalova-Hogenova, Miloslav Kverka

**Affiliations:** ^1^ Laboratory of Cellular and Molecular Immunology, Institute of Microbiology of the Czech Academy of Sciences, Prague, Czechia; ^2^ Laboratory of Gnotobiology, Institute of Microbiology of the Czech Academy of Sciences, Novy Hradek, Czechia; ^3^ Laboratory of Animal Evolutionary Biology, Department of Zoology, Faculty of Science, Charles University, Prague, Czechia; ^4^ Department of Neurology and Centre of Clinical Neuroscience, First Medical Faculty, Charles University and General Medical Hospital in Prague, Prague, Czechia

**Keywords:** multiple sclerosis, experimental autoimmune encephalomyelitis, inflammation, *Parabacteroides distasonis*, microbiota, regulatory T cells

## Abstract

The gut microbiota influences the reactivity of the immune system, and *Parabacteroides distasonis* has emerged as an anti-inflammatory commensal. Here, we investigated whether its lysate could prevent severe forms of neuroinflammation in experimental autoimmune encephalomyelitis (EAE) in mice and how this preventive strategy affects the gut microbiota and immune response. Lysate of anaerobically cultured *P. distasonis* (Pd lysate) was orally administered to C57BL/6 mice in four weekly doses. One week later, EAE was induced and disease severity was assessed three weeks after induction. Fecal microbiota changes in both vehicle- and Pd lysate-treated animals was analyzed by 16S V3–V4 amplicon sequencing and qPCR, antimicrobial peptide expression in the intestinal mucosa was measured by qPCR, and immune cell composition in the mesenteric and inguinal lymph nodes was measured by multicolor flow cytometry. Pd lysate significantly delayed the development of EAE and reduced its severity when administered prior to disease induction. EAE induction was the main factor in altering the gut microbiota, decreasing the abundance of lactobacilli and segmented filamentous bacteria. Pd lysate significantly increased the intestinal abundance of the genera *Anaerostipes*, *Parabacteroides* and *Prevotella*, and altered the expression of antimicrobial peptides in the intestinal mucosa. It significantly increased the frequency of regulatory T cells, induced an anti-inflammatory milieu in mesenteric lymph nodes, and reduced the activation of T cells at the priming site. Pd lysate prevents severe forms of EAE by triggering a T regulatory response and modulating T cell priming to autoantigens. Pd lysate could thus be a future modulator of neuroinflammation that increases the resistance to multiple sclerosis.

## 1 Introduction

Multiple sclerosis (MS) is a chronic autoimmune disease affecting the central nervous system (CNS), which is the leading cause of neurological disability in young adults (1). Several genetic factors drive the immune response in MS (2, 3), but environmental factors e.g., gut microbiota composition, Epstein-Barr virus (EBV) infection, vitamin D deficiency, smoking, obesity, and stress, are important disease triggers (4, 5). Patients with MS have a reduced abundance of Bacteroidetes and Clostridia clusters XIVa and IV and an increased abundance of Akkermansiaceae and Methanobacteriaceae in their stool (6–8). Patients with MS have also reduced abundance of *Parabacteroides* spp (9)., similar to patients with other diseases, such as inflammatory bowel disease (10), psoriasis (11), ankylosing spondylitis (12), or metabolic syndrome (13). The microbiota is an important modulator of an immune response, but little is known about the molecular mechanisms underlying the immune response to microbiota in patients with MS.

Experimental autoimmune encephalomyelitis (EAE) is a widely used animal model of autoimmune inflammatory diseases of the CNS, resembling MS. Mice reared in different animal facilities develop EAE with different disease severity (14), suggesting that gut microbiota can influence the development of the neuroinflammation in EAE. Interestingly, germ-free (GF) mice have an increased proportion of regulatory T cells (Tregs) in the spleen or draining lymph nodes after disease induction and are more resistant to EAE than conventional mice (15). Ex-GF mice colonized with the gut microbiota of patients with MS have higher severity of EAE compared to mice that received the microbiota from healthy donors or to GF mice. One of the most significant differences between these two sets of gut microbiota was marked reduction of *Parabacteroides distasonis* in the gut microbiota of MS patients (9).

*P. distasonis* belongs to the order Bacteriodales, a group of gram-negative anaerobic bacteria that frequently colonize the gastrointestinal tract (16). It induces antimicrobial resistance in other microbes and modulates the T cell immunity by its surface glycoprotein antigens and its metabolites (acetic and succinic acid) (17). *Parabacteroides* species are also well-known inducers of antimicrobial peptides such as Reg3β and Reg3γ (18, 19). Interestingly, Pd extracts are able to induce a significant increase of CD25^+^ T lymphocytes within the CD3^+^CD4^+^ T cell population, with an enrichment of CD25^+^IL-10^+^ cells, including CD25^+^IL-10^+^FoxP3^−^ Tr1 cells but not CD25^+^FoxP3^+^ Treg cells in peripheral blood mononuclear cells from healthy human donors (9). Furthermore, C57BL/6 mice monocolonized with Pd have markedly increased FoxP3^+^ Treg cells in their colonic mucosa (20), and oral treatment with Pd lysate antigens significantly increase CD4^+^CD25^+^FoxP3^+^ cells in mesenteric lymph nodes of mice with dextran sodium sulfate (DSS)-induced colitis (21).

Our previous results have shown that even killed commensal or probiotic bacteria can reduce the severity of intestinal inflammation by modulating immune system activation or gut barrier function (21, 22). Here, we study whether this protective effect can also extend beyond the intestinal inflammation and whether this administration affects the microbiota composition, gut integrity, or T-cell response.

## 2 Materials and methods

### 2.1 Preparation of bacterial lysate of *Parabacteroides distasonis*

Lysate of *Parabacteroides distasonis* was prepared as described previously (21). Briefly, bacterial culture was grown for 24 hours on Wilkins-Chalgren Anaerobe Agar (Oxoid, Basingstoke, UK) enriched with defibrinated blood. Then, the culture was placed into Brain Heart Infusion Broth (Oxoid) for 48 h at 37°C in an anaerobic atmosphere. The cells were harvested by centrifugation (4,000 × g, 30 min) and washed twice with sterile phosphate-buffered saline (PBS). After the disruption with the French Press, the lysate was freeze-dried and diluted to a working concentration of 30 g/l. For membranous fraction preparation, part of the lysate was separated by centrifugation (8,500 × g, 30 min). The insoluble part of the lysate was called membranous fraction. To kill all remaining viable bacteria, the lysate was heated to 60°C for 30 min and the sterility of all components was verified by both aerobic and anaerobic 48 h cultivation before administration (22).

### 2.2 Animals

Female wildtype C57BL/6 mice (8–10 weeks old) were purchased from the breeding colony of the Institute of Microbiology of the CAS. Mice were fed with a Maintenance diet for rats and mice No. 1324 (Altromin Spezialfutter, GmbH & Co. KG, Lage, Germany). Mice were given access to food and water *ad libitum* and kept on a 12 h light/dark cycle. This study was carried out in accordance with the recommendations of the ethics standards defined by the EU legislation on the use of experimental animals (2010/63/EU) and the Czech Animal Welfare Act. The protocol was approved by the Institute of Microbiology Animal Care and Use Committee (approval ID: 29/2020 and 1/2021).

### 2.3 Study design and induction of EAE and assessment of EAE severity

To analyze the effect of bacterial components of *Parabacteroides distasonis*, we administered 1.5 mg of whole lysate or its membranous fraction in 50 µl of sterile PBS by oral gavage as described previously (21). To reduce proteolytic activity in the gut, the Pd components were co-administered with 1 mg of soybean trypsin inhibitor (Sigma-Aldrich, St. Louis, MO, USA) dissolved in 50 µl of 0.15 M sodium bicarbonate buffer (pH 8.0). Control mice were given only sterile PBS with soybean trypsin inhibitor in bicarbonate buffer. The preventive administration of Pd components was repeated every 7 days for a total number of 4 doses (on days 0, 7, 14, and 21) as described previously (21). To analyze the effect of bacterial components of *Lacticaseibacillus casei* DN-114 001 we administered lysate of this bacterium in the same preventive scheme as Pd described above or as described previously (22). Experimental autoimmune encephalomyelitis (EAE) was induced on day 28 and developed until day 46. Mice were immunized by two subcutaneous injections on both flanks, each containing 50 µg of myelin oligodendrocyte glycoprotein peptide MOG_35-55_ (ProSpec-Tany Techno Gene, Ness-Ziona, Israel) emulsified in 50 µL complete Freund’s adjuvant, supplemented with 5 mg/mL of heat-inactivated *Mycobacterium tuberculosis* H37Ra (Sigma-Aldrich). Mice also received two doses of 300 ng pertussis toxin (List Biological Laboratories, Inc., Campbell, CA, USA) intraperitoneally on the day of immunization and 24 hours later (23). Clinical signs of disease (EAE score) were monitored as described, based on progressive deterioration of motor skills (from mild to total paralysis), using the following staging criteria: 0, no clinical signs; 0.5, partially limb tail; 1, flaccid tail; 2, hind limb weakness; 2.5 one hind limb paralyzed; 3, complete hind limb paralysis or one hind and one front leg; 3.5 hind limbs paralyzed and weakness in forelimb; 4, limp tail, complete hind limb paralysis + forelimb paralysis; 5, total paralysis, spontaneous rolling in the cage or deceased (23).

### 2.4 Cell preparation and flow cytometry analysis

Single-cell suspensions from mesenteric lymph nodes (mLN), inguinal lymph nodes (iLN), and spleens were prepared as previously described (24). Next, the cells were blocked by normal mouse serum and anti-CD16/CD32 antibody (BioLegend, Inc., San Diego, CA, USA), and incubated with fluorochrome-conjugated antibodies recognizing extracellular epitopes (**Supplementary Table 1**). To distinguish viable and dead cells, Fixable Viability Dye-eFluor 780) was added. Then, the cells were treated with eBioscience™ Foxp3/Transcription Factor Staining Buffer Set (Thermo Fisher Scientific, Waltham, MA, USA) and stained for intracellular antigens. Data were obtained by measuring the samples on LSRII (BD Biosciences, CA, USA) and analyzed by the FlowJo software (Tree Star Inc., Ashland, OR, USA). An example of used gating strategies is shown in **Supplementary Figures 1**-**5**.

### 2.5 Cell cultures and cytokines measurement

Single-cell suspensions from mLN were cultivated and cytokine production was measured as previously described (25). Briefly, cells were seeded at 2 × 10^5^ live cells per well in 96-well plates in RPMI 1640 (Sigma-Aldrich) culture medium supplemented with 10% fetal bovine serum (BioClot GmbH, Aidenbach, Germany) and 1% Antibiotic-Antimycotic solution (Sigma-Aldrich). Then, the cells were stimulated for 48 hours with plate-bound anti-CD3ϵ (5 μg/ml; clone 145-2C11) and soluble anti-CD28 (2 μg/ml; clone 37.51; both eBioscience) antibodies. The supernatants were collected and frozen at -20°C until analysis. Commercially available ELISA sets were used to measure the levels of IL-10 and IFN-γ in the supernatants (R and D Systems, Minneapolis, MN, USA). All tests were performed according to the manufacturer’s recommendations.

### 2.6 Indirect enzyme-linked immunosorbent assays

The serum concentrations of anti-Pd lysate antibodies of Immunoglobulin M (IgM), Immunoglobulin G (IgG) and Immunoglobulin A (IgA) isotypes were analyzed by in-house developed indirect ELISA exactly as described previously (21).

### 2.7 Quantitative PCR

At the end of the experiment, RNA was isolated from the colon and ileum of control mice, mice with induced EAE, and mice with induced EAE treated with Pd lysate using TRIzol reagent (Invitrogen, Carlsbad, CA, USA). Total RNA (1 µg) was treated with DNAse I (Turbo DNAse; Thermo Fisher Scientific), and reverse-transcribed using the primer Oligo(dT)12–18 and Superscript IV Reverse Transcriptase (Life Technologies, Carlsbad, CA, USA) and subsequently used in a PCR reaction. A web-based tool RefFInder, which uses four different algorithms, was used to select suitable housekeeping genes (https://www.heartcure.com.au/reffinder/) (26). Out of the 5 potential reference genes (ACTB, CasC3, Hprt, Pgk1, S12), we selected a combination of Hprt and Pgk1 as the most stable. The calculated amplification efficiencies for all genes were between 99.2–106.4%. Dilution series of cDNA were analyzed to evaluate amplification efficiency. QPCR (CFX96 Touch™, Bio-Rad Laboratories Inc., Hercules, CA, USA) was performed to determine the changes in mRNA levels of different genes (**Supplementary Table 2**). Cycling parameters were as follows: 4 min at 94°C, 35 cycles of 10 s at 94°C, 25 s at 60°C with a final extension for 7 min at 72°C. Changes in gene expression were calculated using the 2^−ΔΔCT^ (Livak) method (27). The change in mRNA level was related to the change in the established controls. All parameters were measured in duplicates. To obtain a normal distribution, the data were transformed using Boxcox transformation with a lambda value of 0.1. Data were analyzed by one-way ANOVA with Tukey’s multiple comparison test using GraphPad Prism software (version 8.4.3, San Diego, CA, USA). Data results are expressed as mean ± SD.

To determine the abundance of different bacterial groups or species, qPCR (CFX96 Touch™, Bio-Rad) was performed on isolated gDNA from stool samples of mice of all studied groups during the 3 intervals (day 0, day 28 - after pretreatment with Pd lysate, day 46 - end of the experiment). 40 ng of isolated gDNA (as described below in Gut microbiota analysis) were used in qPCR with universal (all bacteria) or species-specific primer sets (**Supplementary Table 2**). Analysis of bacterial abundance between control and treated samples was performed on transformed data (Box-Cox transformation with lambda 0.1) using one-way ANOVA with Tukey’s multiple comparison test. Changes in bacterial abundance between intervals in each group were performed using RM one-way ANOVA with Tukey’s multiple comparison test (GraphPad Prism software).

### 2.8 Gut microbiota analysis

Analysis of gut microbiota was done as previously described (28). The DNA was isolated from the stool specimen using the ZymoBIOMICS DNA Miniprep Kit (Zymo Research, Irvine, CA, USA) according to the manufacturer’s protocol. The V3 and V4 regions of bacterial 16S rRNA gene amplified by specific primers with barcodes (341F GTCCTACGGGNGGCWGCAG and 806R GGACTACHVGGGTWTCTAAT), were chosen as representative sequences for taxonomic identification. The amplification reaction was performed with the KAPA HiFi HotStart Ready Mix (Roche, Penzberg, Germany), as follows: initial denaturation step 3 min at 95°C followed by 25 cycles at 95°C for 30 s, 55°C for 30 s, 72°C for 30 s with a final elongation step at 72°C for 5 min using 5 ng/µL DNA. PCR products were checked using QIAxcel advanced capillary electrophoresis (QIAgen, Hilden, Germany). Triplicates of the amplicons were pooled and normalized with the SequalPrep™ Normalization Plate Kit (Thermo Fisher Scientific), concentrated (Eppendorf centrifugal vacuum concentrator), and purified with DNA Clean & Concentrator Kit (Zymo Research). Subsequently, the amplicon libraries were ligated with sequencing adapters using the KAPA HyperPlus Kit (Roche), pooled in equimolar concentrations, and sequenced. Amplicon sequencing was performed using the MiSeq platform (Illumina, San Diego, CA, USA).

#### 2.8.1 Sequencing data processing and statistics

In the first step sample demultiplexing, primer detection, and trimming were performed using Skewer (29). Low-quality reads (expected error rate per paired-end read > 2) were then eliminated. DADA2 was used to denoise the quality-filtered reads and quantify 16S rRNA Amplicone Sequence Variants (hereafter, ASVs) in each sample (30). Chimeric ASVs were detected and eliminated using UCHIME and gold.fna reference database (https://drive5.com/uchime/gold.fa) (31). The taxonomy assignation of non-chimeric ASVs was conducted using the RDP classifier with 80% confidence threshold and the Silva database 138 as a reference (32, 33). Chloroplasts and mitochondria, as well as sequences that could not be assigned to a bacterial phylum, were considered contaminants of the diet or sequencing artefacts and were excluded from all downstream analyses. Sequences from technical duplicates were merged for each sample. The ASV abundance matrix (i.e. the number of ASV reads in each sample), ASV sequences, their taxonomic annotations and phylogeny were merged into a single database along with sample metadata using the phyloseq package in R (R Core Team 2020) (34). Sequencing data are available at European Nucleotide Archive (ENA). To standardize sequencing depth, we rarefied the ASV table for alpha and beta diversity analysis. Rarefaction depth was set to a sample size of the minimal sequencing depth where most species have been observed within a given number of samples. The alpha diversity was expressed as Shannon diversity index and compared using the Kruskal-Wallis test followed by Dunn’s test for multiple comparisons. Principal Coordinate Analysis (PCoA) was performed to investigate differences in microbiota composition between samples. We used relative ASV abundance (Bray-Curtis index; Bray and Curtis 1957) (35, 36). To evaluate the differences in beta diversity between groups, we used Permutational Multivariate Analysis of Variance Using Distance Matrices (PERMANOVA) using a vegan package (37). Taxonomical analysis was performed using the microViz package, relative abundances were calculated and presented (38). Visualization of the microbial composition by the heatmap was performed using the centered-log-ratio transformation of taxa at the genus level. Ggplot2 package was used for the graphical visualization of the data (39).

## 3 Results

### 3.1 Pd lysate prevents EAE development

**Figure 1:**
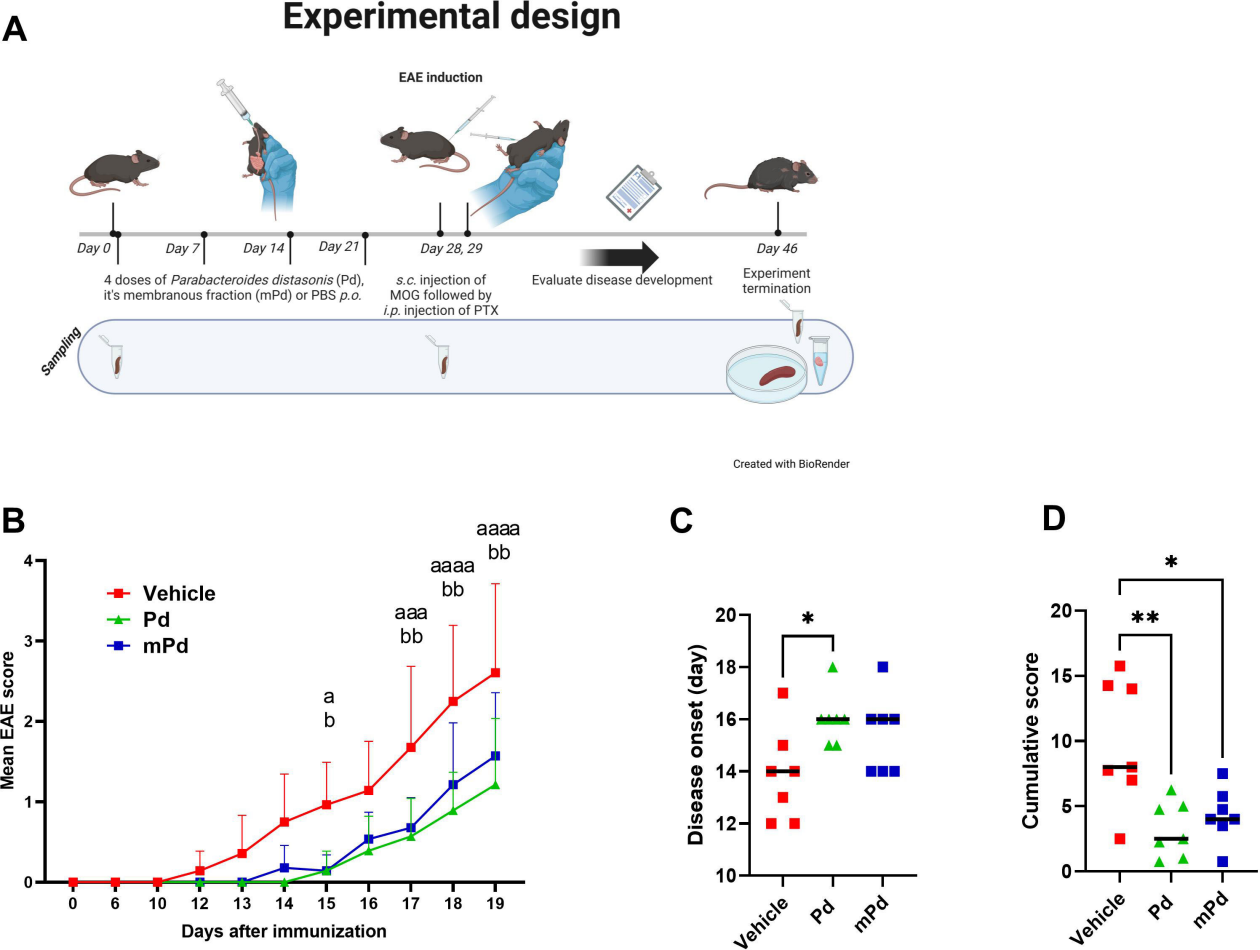
Pd lysate delayed the onset of EAE and decreased its severity. **(A)** Experimental design **(B)** Clinical disease scores. **(C)** Disease onset was defined as the first day a mouse displayed any symptoms. **(D)** The Cumulative score depicts the sum of EAE scores over time for each mouse. The data are representative of one out of four independent experiments (n = 7 mice per group). Statistical significance was determined by ANOVA. *p < 0.05; **p < 0.01. Experimental design was created with BioRender.com/J27DFVAX4. ^a^p < 0.05; ^aaa^p < 0.001 ; ^aaaa^p < 0.0001 or ^b^p , 0.05; ^bb^p < 0.01. ^a^ refers to the comparison of groups Vehicle and Pd; ^b^ refers to the comparison of groups Vehicle and mPd; Pd-lysate of *Parabacteroides distasonis*; mPd - membranous fraction of *Parabacteroides distasonis*.

To investigate the effect of Pd lysate on the development of EAE, we treated mice with four weekly oral doses of either the whole Pd lysate or its membranous fraction (**Figure 1A**). We found that both treatments delayed the onset of EAE and reduced its severity (**Figures 1B–D**). To test whether this is specific to Pd, we performed a similar experiment with the lysate of the anti-inflammatory probiotic *Lacticaseibacillus casei* DN-114 001. While this treatment was similarly effective as Pd in attenuating DSS-induced colitis in our previous experiments (22), it failed to prevent severe forms of EAE (**Supplementary Figure 6**).

### 3.2 Pd lysate modulates the composition of the microbiota before and after EAE induction

**Figure 2:**
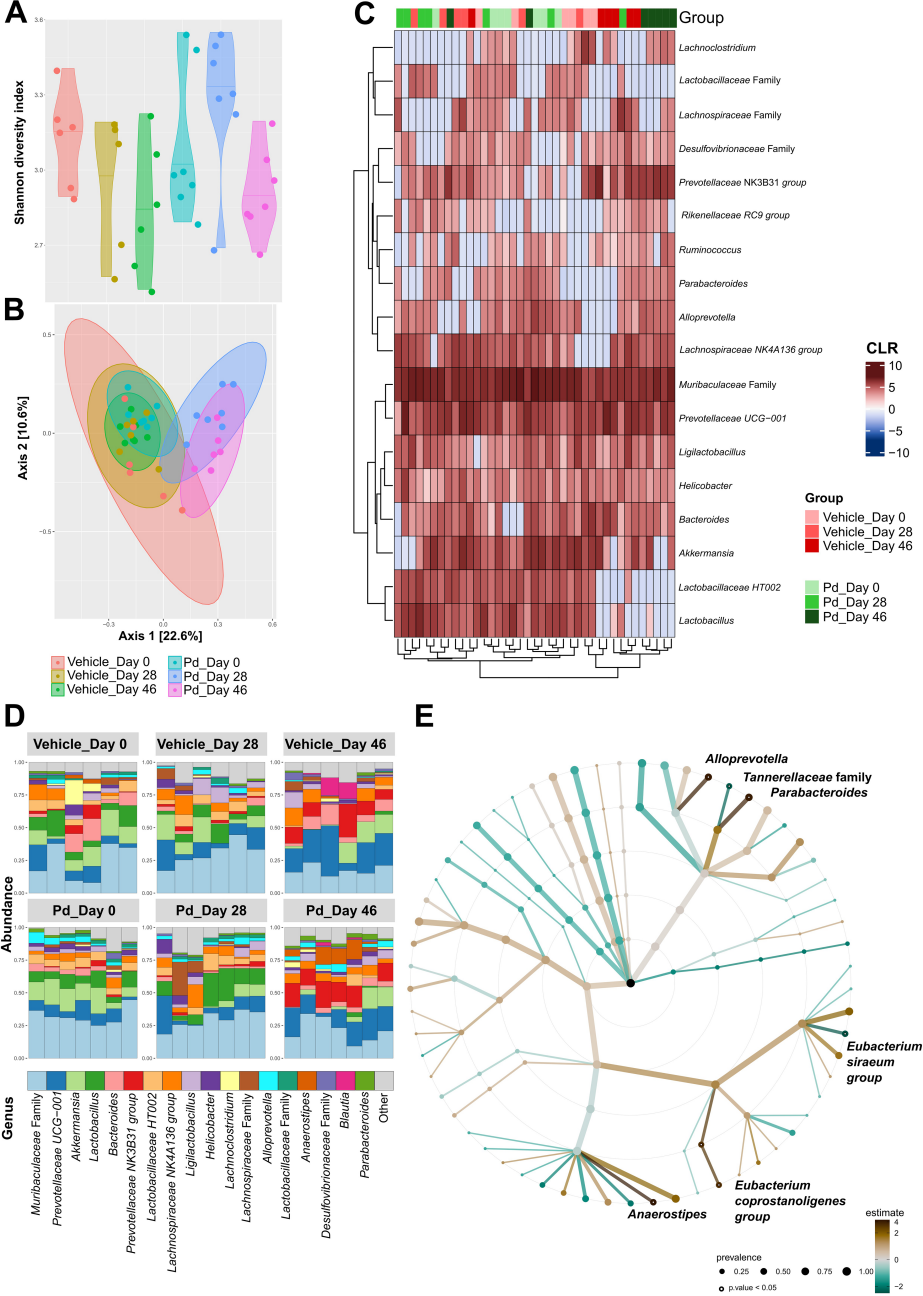
Pd lysate administration changes the microbiota composition before and after EAE induction. **(A)** Alpha diversity was assessed using the Shannon diversity index. The significant differences among groups were tested using the Kruskal-Wallis test followed by Dunn’s test for multiple comparisons. **(B)** Beta diversity was evaluated using Bray-Curtis dissimilarities and Principle Coordinate Analysis (PCoA) where each point represents one mouse and tested with PERMANOVA. **(C)** A heatmap with a depicted centered log ratio (CLR) accompanied by the clustering analysis. **(D)** Relative abundances of the top 18 most abundant taxa of gut microbiota at the genus level are presented as bar plots for each group. **(E)** The statistical differences in abundances of genera between the groups (vehicle and Pd group at day 46 of the experiment termination) were estimated using the TSS log2 linear regression and is presented as a taxonomical tree for a dedicated time point. Only the significantly different genera are depicted. The graphs show the results of one representative experiment (n = 6–7 mice per group).

**Figure 3:**
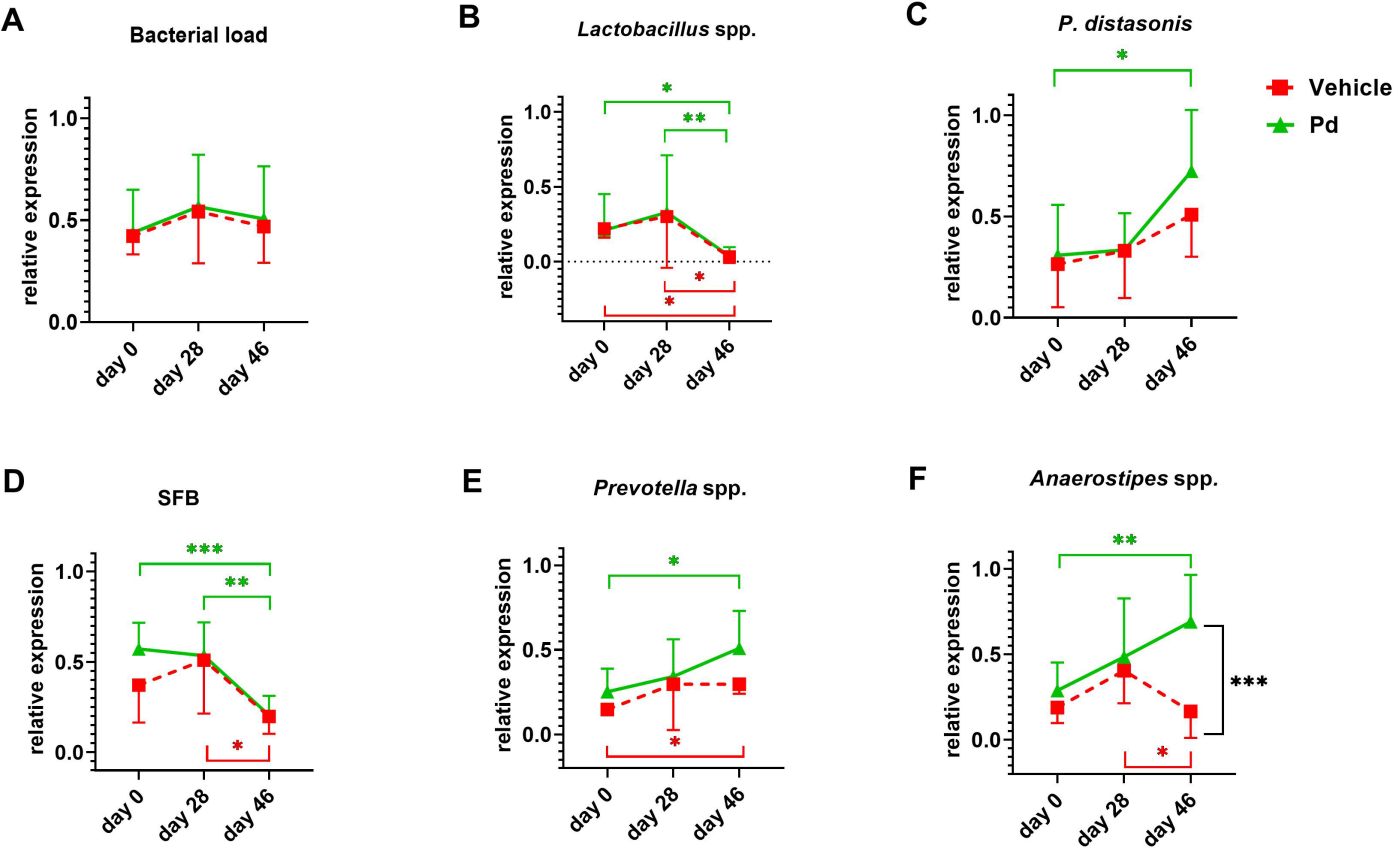
Relative frequencies of selected bacterial specieas and bacterial counts. Relative frequencies of total bacterial count **(A)**, *Lactobacillus* spp. **(B)**, *P. distasonis* **(C)**, segmentous filamentous bacteria (SFB) **(D)**, *Prevotella* spp.**(E)**, and *Anaerostipes* spp. **(F)** in stool samples using RT-qPCR. Stool samples were collected in both groups at 3 intervals – at the beginning of the experiment (day 0), after pretreatment with Pd lysate or vehicle (day 28), and at the end of the experiment (day 46). The relative expression of bacterial genes for 16S rRNA is related to the amount of 40 ng gDNA in a RT-qPCR reaction. Statistical significance between intervals was determined by repeated measures one-way ANOVA, and differences between treatments were assessed by one-way ANOVA with Tukey’s multiple comparison test. *p < 0.05; **p < 0.01; ***p < 0.001;(n = 5–7 mice per group).

Germ-free mice are resistant to EAE induction (15), suggesting that the gut microbiota is a potent modulator of the inflammatory immune response. To analyze how oral Pd lysate administration alters the composition of the microbiota during EAE development, we collected feces before treatment (day 0 - beginning), before EAE induction (day 28 - induction), and at the end of the experiment (day 46 - termination). We found that treatment with Pd lysate did not significantly changed microbial alpha diversity (**Figure 2A**), but significantly altered the composition of intestinal microbiota (**Figures 2B–D**). Although the induction of EAE seems to be the main trigger for microbial shifts, the difference between vehicle and Pd was still clearly recognizable even after the induction of EAE (**Figures 2B–D**). Interestingly, Pd-treated mice had a significantly higher abundance of the genus *Parabacteroides* in their stool than PBS-treated mice at the time of termination (**Figure 2E**). Prior to EAE induction, there were no differences between these groups in terms of *Parabacteroides* spp. abundance. In addition, Pd lysate administration significantly increased abundance of the genera *Anaerostipes*, *Eubacterium* (coprostanoligenes group/family) and *Alloprevotella*, and decreased family *Tanerellaceae* in mice with EAE but not in the control group (**Figure 2E**; **Supplementary Figure 7**). To further quantify the abundance of the different bacterial groups or species we performed qPCR analysis. We further confirmed that EAE induction reduces the abundance of *Lactobacillus* spp. in the feces of mice either with or without pretreatment with Pd (**Figure 3B**). The induction of EAE led to a significant increase in the abundance of *P. distasonis*, which was no longer present after the administration of Pd lysate (**Figure 3C**). The abundance of SFB was significantly reduced in the Pd lysate group after EAE induction (**Figure 3D**). A significantly increased abundance of *Prevotella* spp. and *Anaerostipes* spp. was observed in the Pd-treated group, which gradually increased throughout the experiment (**Figures 3E, F**). The abundance of *Anaerostipes* spp. differed significantly between vehicle and Pd at the end of the experiment and the abundance of SFB decreased in the Pd-treated group. Similar trends were observed in the PBS-treated animals, but these differences were not statistically significant. There were no significant differences between vehicle and Pd-treated animals at the same time point and neither EAE nor Pd treatment changed the bacterial load (**Figure 3A**).

### 3.3 Pd changes the local production of antimicrobial peptides

**Figure 4:**
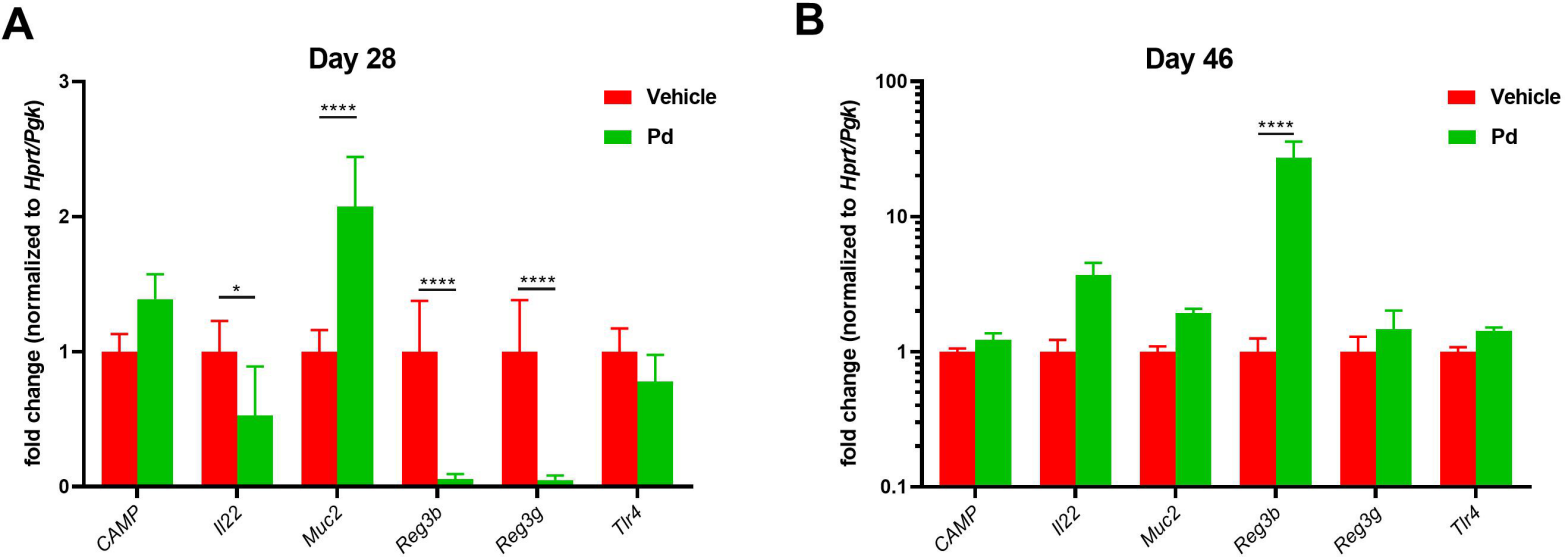
Pd lysate decreases Il22, Reg3b and Reg3g expression and increases Muc2 expression at the time of induction. Colonic expression of CAMP, Il22, Muc2, Reg3b, Reg3g and Tlr4 between PBS- and Pd-treated animals both at the EAE induction (day 28) **(A)** and termination (day 46) **(B)**. Data presented are from one experiment out of three, n = 5–8 mice per group. Means significantly different from vehicle were calculated by one-way ANOVA. Data are shown as mean ± SD. *p < 0.05; ****p < 0.0001. EAE, experimental autoimmune encephalomyelitis; Hprt, hypoxanthine guanine phosphoribosyl transferase; Pgk, phosphoglycerate kinase 1; CAMP, Cathelicidin antimicrobial peptide; Il22, Interleukin 22; Muc2, Mucin 2; Reg3b, regenerating islet-derived protein 3 – beta; Reg3g, regenerating islet-derived protein 3 – gamma; Tlr4, Toll like receptor 4.

Antimicrobial peptides (AMP) and mucins can shape the gut microbiota composition (40). Therefore, we compared AMP expression between PBS- and Pd-treated animals both at the EAE induction (day 28) and termination (day 46) (**Figures 4A, B**). We found that Pd decreases Il22, Reg3b and Reg3g expression and increases Muc2 expression at the time of induction (**Figure 4A**). However, the combined effect of Pd and EAE erased this profile, leading to increased expression of Reg3b in Pd-treated animals at termination (**Figure 4B**).

### 3.4 Induction of EAE leads to subtle damage to the mouse intestine and Pd lysate shifts the substantial local immune response to an anti-inflammatory tuning

**Figure 5:**
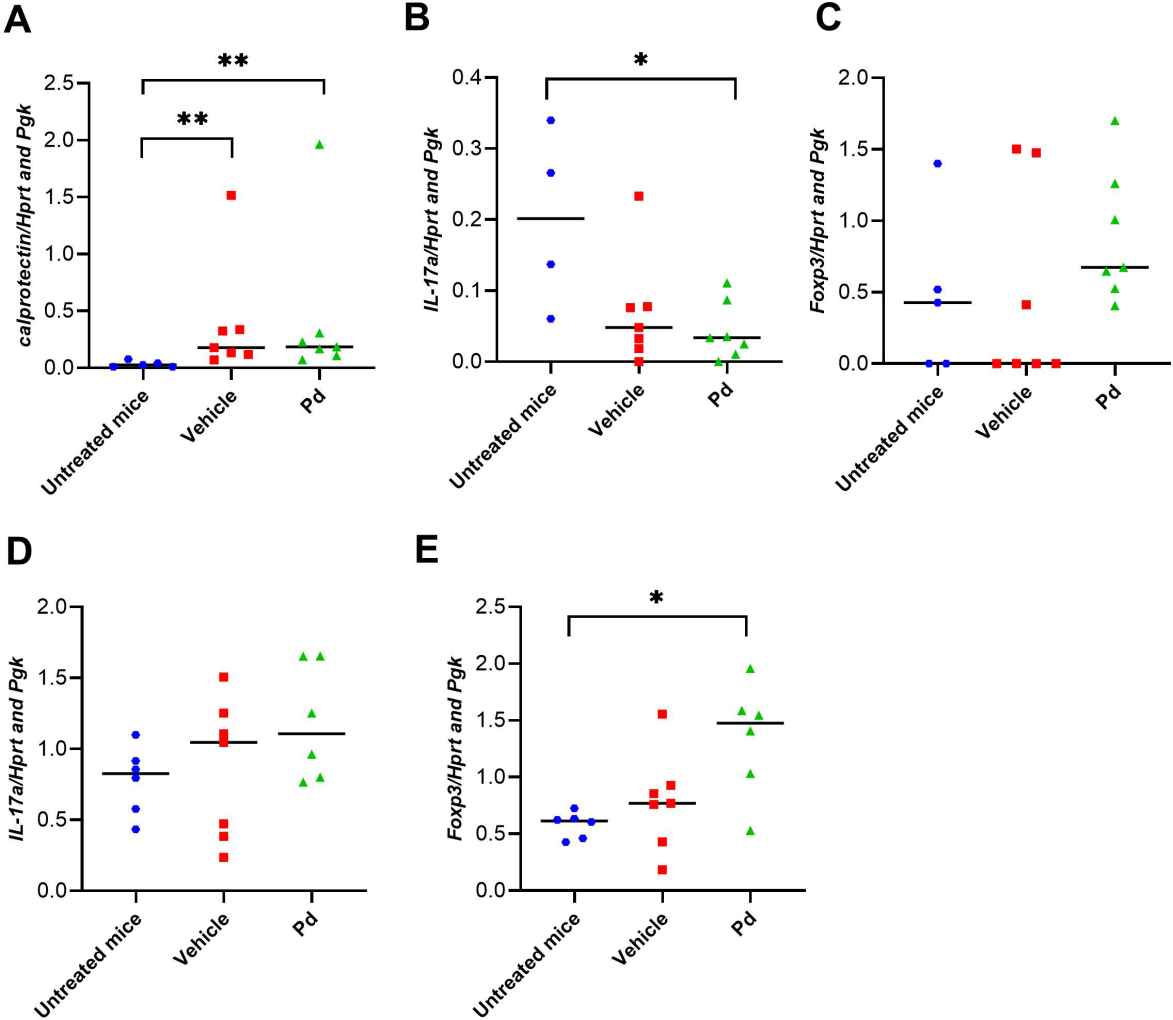
Pd lysate modulates the immune system in the intestine. Colonic expression of calprotectin **(A)**, Il17a **(B)**, Foxp3 **(C)**, in healthy mice, EAE-induced mice (vehicle), and EAE-induced mice treated with Pd lysate (Pd) at the day 46. Statistical significance was determined by ANOVA. *p < 0.05; **p < 0.01; (n = 5–7 mice per group). Ileum gene expression of Il17a **(D)**, Foxp3 **(E)**, in healthy mice, EAE-induced mice (vehicle), and EAE-induced mice treated with Pd lysate (Pd). EAE, experimental autoimmune encephalomyelitis; FoxP3, forkhead box P3; HPRT, hypoxanthine guanine phosphoribosyl transferase; IL-17 – interleukin 17 Pgk, phosphoglycerate kinase 1.

**Figure 6:**
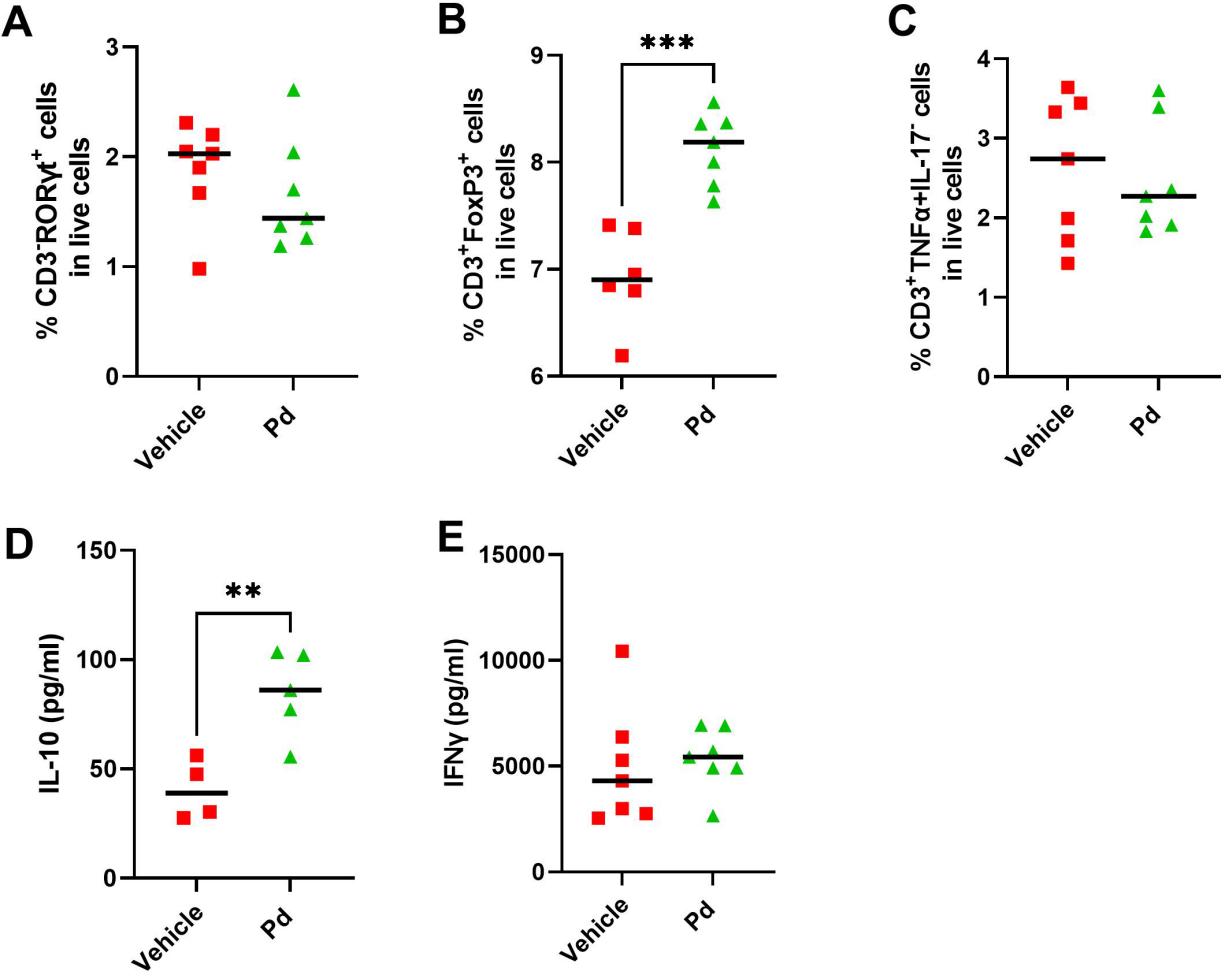
Pd lysate modulates the immune system in the mesenteric lymph nodes (mLN). Flow cytometry analysis of mLN T cell staining for Th17 cells (CD3^+^RORγt^+^) **(A)**, Treg cells (CD3^+^Foxp3^+^) **(B)** and CD3^+^TNF-α^+^IL-17^-^T cells **(C)**. Production of IL-10 **(D)** and IFN-γ **(E)** in mLN after anti-CD3/CD28 stimulation. Statistical significance was determined by unpaired Student’s t-test. **p < 0.01 ***p < 0.001;(n = 5–7 mice per group).

Dysbiosis associated with altered AMP production could lead to subtle changes in intestinal mucosal integrity and subsequent stimulation of the intestinal mucosal immune system by luminal antigens. Therefore, we investigated whether the protective effect of Pd lysate in the mouse model of EAE is associated with changes in the inflammatory response in the colon and ileum at EAE termination (Day 46). First, we quantified the damage to the intestinal mucosa by measuring the expression of the inflammatory marker calprotectin. Both groups of mice with induced EAE had significantly increased calprotectin expression in the colon compared to healthy mice regardless of Pd treatment, indicating colonic mucosal damage and inflammation (**Figure 5A**). Since a more permeable intestinal mucosa and local inflammation could alter the local T-cell balance, we also measured the expression of Il17a and Foxp3 in the colon and ileum. Compared to healthy mice, Il17a expression in the colon but not ileum was downregulated in both groups of EAE mice (vehicle-treated mice showed a strong but non-significant trend) (**Figures 5B, D**). In the colon, there were no significant differences in Foxp3 expression between the groups, with a slight tendency toward upregulation in the Pd-lysate-treated group (**Figure 5C**). However, the expression of Foxp3 was significantly increased in the Pd-lysate-treated group compared to the untreated mice (**Figure 5E**). To further analyze the immunomodulatory effect of Pd lysate, we measured the cytokine production and Treg numbers in mesenteric lymph nodes (mLN). We found that Pd lysate decreased proportion of type 3 innate lymphoid cells ILC3 (CD3^-^RORγt^+^) (**Figure 6A**), significantly increased the Treg proportion (**Figure 6B**), but did not influence the proportion of T cells to producing pro-inflammatory TNF-α (**Figure 6C**). Pd lysate significantly increased ability of T cells to produce anti-inflammatory IL-10 and did not alter the production of IFN-γ upon their TCR stimulation *ex vivo* (**Figures 6D, E**). Pd-lysate modulates the immune response of the mesenteric lymph nodes, which could spread through the body and influence the severity of EAE via the gut-brain axis. To follow the ability of Pd lysate to cross the intestinal barrier in the EAE model, we additionally analysed the specific IgG, IgM and IgA antibody response to Pd lysate in serum. We did not detect increased IgG, IgM and IgA levels against Pd lysate in the Pd lysate group (**Supplementary Figure 9**).

### 3.5 Oral administration of Pd lysate modulates immune response in distant lymph nodes

**Figure 7:**
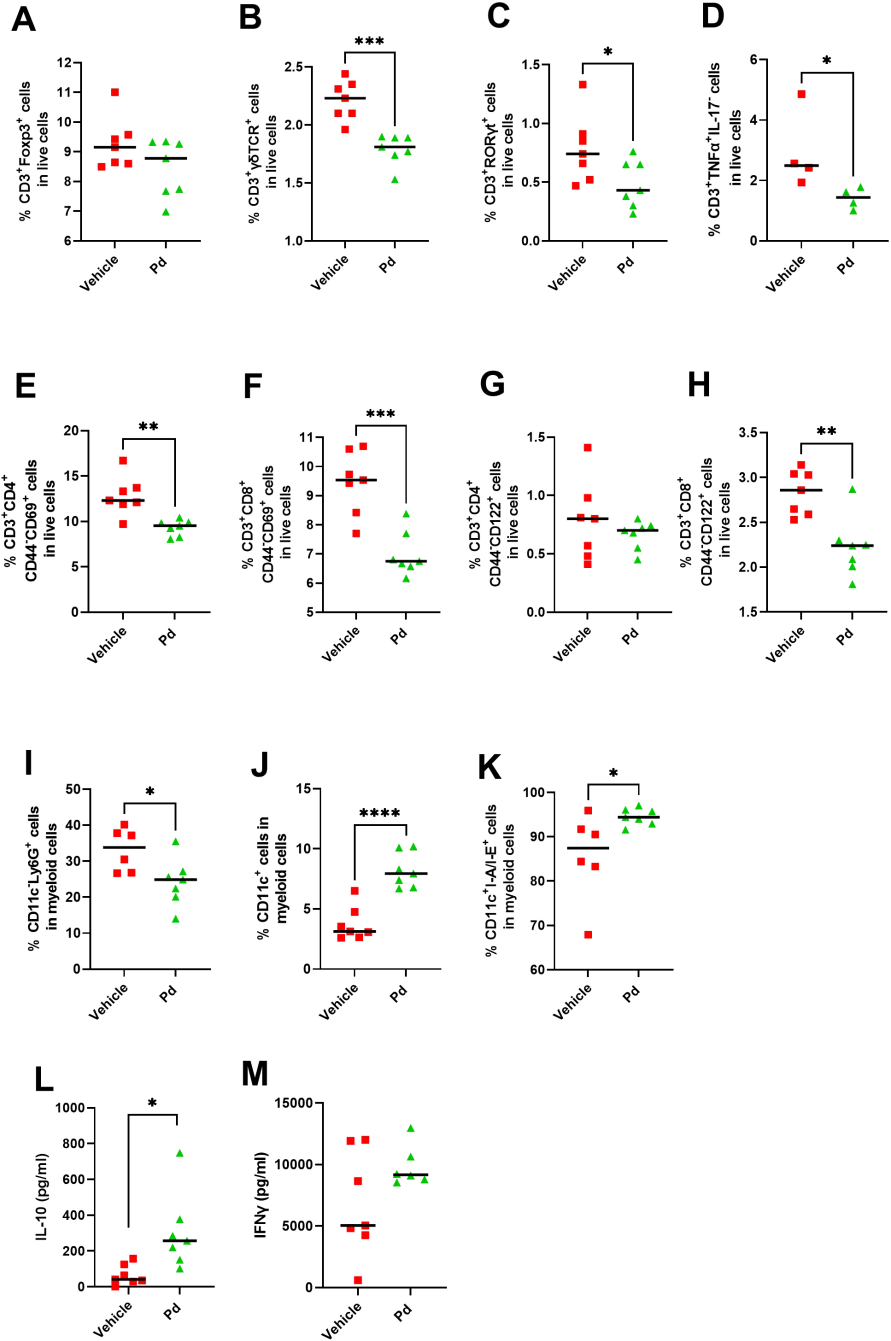
Treatment with Pd lysate shift in T cell phenotype and alters the subsets of innate immune cells in distant inguinal lymph nodes (iLN). Flow cytometry analysis of iLN for Treg cells (CD3^+^Foxp3^+^) **(A)**, CD3^+^γδTCR^+^ **(B)**, CD3^+^RORγt^+^ **(C)**, CD3^+^TNF-α^+^IL17^−^ **(D)**, for T helper cells *(*CD3^+^CD4^+^CD44^−^CD69^+^) **(E)**, cytotoxic T cells (CD3^+^CD8^+^CD44^−^CD69^+^) **(F)**, CD3^+^CD4^+^CD44^−^CD122^+^ **(G)**, CD3^+^CD8^+^CD44^−^CD122^+^ cells **(H)**, for neutrophils (CD11c^−^Ly6G^+^) **(I)**, dendritic cells (CD11c^+^) **(J)** and their maturation state (CD11c^+^I-A/I-E^+^) **(K)**. Production of IL-10 **(L)** and IFN-γ **(M)** in iLN after anti-CD3/CD28 stimulation. The analyses were performed on day 46 and data are representative of one out of three independent experiments (n = 5-7 mice per group). Statistical significance was determined by unpaired Student’s t-test *p < 0.05; **p < 0.01, ***p < 0.001, ****p < 0.0001.

Next, we investigated whether the anti-inflammatory effect of oral Pd lysate during EAE induction can spread to the lymph nodes draining the original site of antigen deposition. Therefore, we analyzed the phenotype of the cells involved in the antigen presentation, T cells and dendritic cells, in the iLN at EAE termination (Day 46). We found that while Pd lysate did not increase the proportion of regulatory T cells (CD3^+^Foxp3^+^) in iLN (**Figure 7A**), it decreased the proportion of CD3^+^γδTCR^+^ T cells (**Figure 7B**). Moreover, it shifted the T cells to more naïve and less activated phenotype, as evidenced by a lower proportion of CD3^+^RORγT^+^ and a lower proportion of T cells producing pro-inflammatory TNF-α compared to PBS treated group (**Figures 7C, D**). Pd treatment decreased the percentage of activated T helper cells (CD4^+^CD44^-^CD69^+^) and cytotoxic T cells (CD8^+^CD44^-^CD69^+^) (**Figures 7E, F**), thus decreasing immediate effector functions in iLN. Pd also decreased CD122^+^ expression on CD3^+^CD8^+^CD44^-^, but not in CD3^+^CD4^+^CD44^-^, T cells (**Figures 7G, H**). Interestingly, mice treated with Pd, had a lower percentage of neutrophils (CD11c^-^Ly6G^high^) (**Figure 7I**) and a higher percentage of dendritic cells (CD11c^+^) out of myeloid cells (CD3^−^CD49b^−^B220^−^) compared to the control group (**Figure 7J**). The total lysate of Pd also led to an increased dendritic cells maturation, which was expressed as an increased number of CD11c^+^I-A/I-E^+^ cells (**Figure 7K**). Similar effects were apparent even if only membranous fraction of Pd was used (**Supplementary Figure 8**), suggesting that these changes are driven mainly by bacterial cell walls and not by the low-molecular-weight metabolites of Pd. Similar to mLN, Pd lysate increases ability of T cells to produce anti-inflammatory IL-10 and did not alter the production of IFN-γ in iLN upon their TCR stimulation *ex vivo* (**Figures 7L, M**). Taken together, oral Pd lysate downregulates the activation state in immune cells in distal iLN, thus attenuating EAE severity.

## 4 Discussion

*Parabacteroides distasonis* is a gram-negative, obligate anaerobic commensal that occurs in both mice and humans. Compared to healthy individuals, it is significantly reduced in the intestines of people suffering from inflammatory diseases such as rheumatoid arthritis or MS (9, 41). When used in mouse models of these diseases, *P. distasonis* showed clear anti-inflammatory properties and significantly prevented the development of these diseases. In these cases, *P. distasonis* promoted anti-inflammatory mechanisms of adaptive immunity, especially regulatory T cells (9, 41). In our previous work, we found that even lifeless Pd lysate induced local T regulatory cells and protected mice from experimental colitis (21). This previously used preventive treatment and the dosages have been extensively validated in acute DSS colitis and since it was able to induce a local increase in Tregs (21), we hypothesize that it may also be useful in EAE. Here, we analyzed if Pd lysate could influence the development of extra-intestinal inflammation in the mouse model of MS, and we analyzed the impact of this treatment on the gut microbial community as well as on local and systemic immune response.

The gut microbiota of MS patients shows a lower abundance of *P. distasonis*, and leads to more severe EAE symptoms when transferred to mice with EAE, compared to fecal bacteria from household controls (9). We found that oral Pd lysate or its crude membranous fraction both delayed and alleviated the symptoms of EAE when Pd lysate was administered before the induction of EAE. This suggests that the key mechanisms involve initial antigen presentation and T cell priming. Achieving a similar effect in therapeutic situations when the Pd lysate is administered after induction of EAE requires further investigation, as manipulation of the gut microbiota may prevent rather than treat ongoing inflammation, as we have already shown for *Escherichia coli* Nissle 1917 in experimental autoimmune uveitis (42). Therefore, the therapeutic benefit of such approaches must be interpreted with caution. We were not able to mitigate EAE with the lysate of anti-inflammatory probiotic *Lacticaseibacillus casei* DN-114 001, which also induces local Tregs and prevents severe forms of acute DSS-colitis (22). This suggests that the effect of *L. casei* lysate may be more effective in preventing intestinal barrier damage that would influence the course of acute colitis, but was less effective in regulating distant autoimmune inflammation.

The gut microbiota shapes the immune response, and its shifts are associated with the pathogenesis of neuroinflammation (4, 9). Lower α-diversity is sometimes found in patients with MS (43), but this finding is not universal and some studies found either no difference or even increased α-diversity in MS patients (44–46). This likely depends on the population studied and the α-diversity metrics used. However, decreased microbial diversity is the most common form of dysbiosis associated with the development of inflammatory diseases (9, 45, 47, 48). Similar to others (49), we also found that the induction of EAE itself significantly altered the composition of the gut microbiota. All groups had similar bacterial load, but mice with EAE tended to have a less diverse gut microbiota regardless of Pd treatment, although these differences were not statistically significant. The Pd lysate altered the gut microbiota, albeit in a more subtle manner than EAE. It significantly increased abundance of the genera *Anaerostipes*, *[Eubacterium] coprostanoligenes group* and *Alloprevotella*, and decreased family Tanerellaceae. Since MS patients have significantly fewer *Parabacteroides* spp., *Prevotella* spp. and *Anaerostipes* spp. in their gut microbiota compared to healthy controls (34–36), treatment with Pd lysate may have an indirect effect via these microbes, which are less abundant in patients with MS. When we quantified these changes by RT-qPCR, we found that EAE induction reduced the abundance of *Lactobacillus* spp. and SFB. This is in agreement with other studies, where abundance of lactobacilli also decreased dramatically after the onset of EAE, similar to patients with increased activity of MS (47, 49). This suggested that lactobacilli may be the driving force of gut microbiota dysbiosis during the onset of EAE, as their decrease correlates with microbial community reorganization. This result is consistent with our previous work in which we were able to downregulate intestinal inflammation by oral administration of *L. casei* lysate, indicating the immunomodulatory capabilities of lactobacilli (22). Furthermore, in an experimental model of skin inflammation, we have shown that treatment of mice with a broad antibiotic mixture leads to reduction in local and systemic Th17 activation and massive changes in the microbiota composition (25). We described that the most striking effect on the composition of the gut microbiota was a rapid increase in the order Lactobacillales and a significant decrease in Coriobacteriales and Clostridiales (25). We found significant increase in abundance of *P. distasonis* during EAE in Pd lysate treated mice and slight trend in vehicle treated mice. Intestinal Treg cells can control the composition of the gut microbiota, and loss of functional Tregs leads to an increase in secretory IgA-coated commensal bacteria and a marked shift in the composition of the gut microbiota (50). Therefore, an increase in gut Tregs in Pd-treated animals may be partially responsible for the increase in the amount of *P. distasonis* in the gut. We can also speculate that the delayed increase in *P. distasonis* abundance has nothing to do with EAE induction, but is a consequence of the additional time required to induce these cells and alter the balance of the gut microbiota. Interestingly, we found similar trend in *P. distasonis* abundance after oral treatment with antigens isolated from Pd lysate in the stool during DSS-induced colitis (21).

The interactions between microbiota and inflammatory response are plausible, but these differences may also be an artifact of the model. Enzyme blockade with SBTI had no effect as there were no major differences between the time of onset and induction in vehicle-treated animals. However, the CFA injection itself was able to alter the gut microbiota without inducing neuroinflammation, which could explain a similar change in gut microbiota in Pd lysate- and vehicle-treated mice (49, 51). This suggests that the changes in the microbiota induced by Pd lysate are not caused by the Pd content but by the induction of changes in the host mucosa, either by antimicrobial peptides or by inflammation.

The intestinal mucosa maintains homeostasis with commensal bacteria through a feedback mechanism involving innate immunity signaling (52). Many antimicrobial compounds are released in the gut and distributed in a mucus layer to protect the mucosa (53). Some microbes can alter the expression of these factors by their structural components, affecting the shape of the colonizing microbial community for a long time after the microbe has disappeared (54). A similar mechanism could be also triggered by the structural components of the Pd lysate. Therefore, we analyzed the effect of Pd lysate on the expression of several potent antimicrobial peptides both at the time of induction and at the time of termination. We found that Pd lysate decreased the expression of Reg3b and Reg3g before EAE induction, but increased Reg3b after EAE induction. Both lectins are quite similar in structure, but Reg3γ targets Gram-positive bacteria, while Reg3β targets mainly Gram-negative bacteria and only some Gram-positive bacteria (55). The pattern of their expression may be responsible for the shifts in alfa diversity of microbiota during EAE induction, as mice treated with Pd lysate tend to have a higher Shannon diversity index prior to EAE induction. This suggests that Pd lysate changes gut microbiota through the shift in intestinal production of antimicrobial peptides. However, the increased calprotectin expression in EAE mice suggests that EAE induction, independent of treatment with Pd lysate, triggers mucosal damage and subsequent subtle intestinal inflammation.

We found that EAE downregulated the expression of colonic *Il17a* and oral Pd lysate induced a significant increase in ileal, but not colonic, *Foxp3* expression. This difference between vehicle and Pd lysate-treated animals is reflected in a significant increase in the percentage of regulatory T cells, and by increased IL-10 and decreased TNF-α production in the mLN of Pd-lysate treated animals. This regulatory environment appears to be quite robust, as we found a similar increase in Tregs in the mLN in our previous work on acute experimental colitis (21). Tregs are important for the control of neuroinflammation, as reduced numbers of Tregs, impaired Treg suppressive capacity, or resistance of pathogenic T cells to Treg control contribute to the dysregulated T cell-mediated autoimmune responses associated with MS pathology (15, 56). Adoptive transfer of MOG-specific Tregs results in a significant reduction in disease severity and less inflammatory infiltration and demyelination in the spinal cord compared to control animals (57). However, we did not analyze the typical pathological changes of CNS, such as degree of demyelination or cell infiltration in the spinal cord. We assume that animals with a poorer clinical score have a higher rate of demyelination and immune cell infiltration in the brain, as this is already well established (58, 59), but we cannot exclude the possibility that oral Pd treatment primarily attenuates only one of these features. Since we found that Pd lysate enhances the intestinal barrier and that serum IgG, IgM and IgA levels against Pd are not elevated, we do not expect translocation of Pd lysate across the intestinal or blood-brain barrier. Our finding that Pd lysate can increase the Treg response has been documented for live *P. distasonis* in both germ-free and conventional mice (9, 20). This suggests that this property is due to the interaction of the structural components of the bacterium. The mechanism behind this finding could be that Pd lysate stimulates the induction of tolerance, thereby preventing autoimmunity. We have shown that Pd lysate induces Tregs in the gut and local lymph nodes and that this correlates negatively with the EAE severity. This suggests that these Pd lysate-induced Tregs may be able to migrate to the site of inflammation and dampen it – either back into the colon in colitis or along the gut-brain axis in EAE (60). In contrast to human MS, the first interaction with the MOG antigen occurs in the iLN draining the site of priming. Nevertheless, we did not investigate the degree of their infiltration into the CNS or tracked their trafficking, so we cannot prove that Pd has the same effect on Tregs in CNS as on Tregs in ileum or mLN and that these cells will migrate into the CNS, which is the main limitation of this study.

We found that Pd treatment increased IL-10 levels in both mLN and iLN without affecting IFN-γ production. This suggests that the anti-inflammatory effect of Pd persists both at the site of interaction with Pd (mLN) and at the site of immunization with MOG_35-55_ (iLN). IFN-γ, a hallmark cytokine of Th1 cells, is thought to drive inflammation in EAE and MS (61, 62). Although it remains an important pathway of immune reactivity, current research now takes a more balanced view of its pathogenic role in EAE, as several neuroprotective effects of IFN-γ have been uncovered (63–65). In fact, IFN-γ may even limit the development of EAE (66–68). This depends on the disease stage, as IFN-γ promotes the induction phase of EAE pathogenesis, while limiting disease progression during the early effector phase (69, 70). Our data suggest that despite its function in EAE, IFN-γ does not play a key role in Pd lysate-induced attenuation of EAE.

We found that treatment with Pd lysate attenuated the proinflammatory activation of the immune system in the iLN by delaying the activation of T lymphocytes. Pd lysate increased the proportion of mature DCc (CD11c^+^MHCII^+^) but decreased the proportion of neutrophils (CD11c^-^Ly-6G^+^), activated T helper cells (CD4^+^CD44^-^CD69^+^) and cytotoxic T cells (CD8^+^CD44^-^CD69^+^) as well as CD8^+^ memory stem T cells (T_SCM_; CD3^+^CD8^+^CD44^-^CD122^+^). However, we did not find any significant difference in the number of Treg cells in iLN. Similarly, as an increase in Tregs, the decrease in CD69^+^ T cells could prevent the homeostatic imbalance of the immune system that could be responsible for the intense autoreactive T cell activity (71). The decrease in T_SCM_ in the Pd lysate-treated mice is interesting because these cells have a high capacity for self-renewal and have been implicated in the pathogenesis of chronic inflammatory responses, such as response to tumors and GVHD (72, 73). The effect of Pd lysate on decreasing neutrophil frequency could also explain a decrease in EAE severity in these mice, as neutrophils are involved in the pathogenesis of MS (74).

## 6 Conclusions

The lysate of *Parabacteroides distasonis* modulates the adaptive immune response in the intestine. This anti-inflammatory effect is even extended to distant lymph nodes, where it prevents the development of a strong autoimmune response to MOG. This prevents the severe forms of neurological impairment in EAE if administered before the initial T cell priming. Thus, the lifeless lysate of *P. distasonis* may have the ability to increase the resilience to MS or support the prolongation of the remission phase in MS patients. Harnessing this ability with a sterile bacterial lysate would be a safer, simpler, and cheaper alternative to live bacteria.

## Data Availability

The datasets presented in this study can be found in online repositories. The names of the repository/repositories and accession number(s) can be found below: https://www.ebi.ac.uk/ena, PRJEB78598.
