# Supplementary material for: Lysate of Parabacteroides distasonis prevents severe forms of experimental autoimmune encephalomyelitis by modulating the priming of T cell response
Source: Front Immunol. 2024 Dec 16;15:1475126. doi: 10.3389/fimmu.2024.1475126 (PMC11682988; doi:10.3389/fimmu.2024.1475126)
Supplement: Supplementary file 1 [file DataSheet1.docx]

Supplementary Material

**Supplementary Table 1.** Fluorochrome-labeled antibodies and stains used for flow cytometry.

| Epitope – Fluorochrome | Clone | | Manufacturer | Cat# | RRID |  |
| --- | --- | --- | --- | --- | --- | --- |
| Fixable Viability Dye eFluor 780 | | - | Thermo Fisher Scientific | 65-0865-14 | - |  |
| CD3e – FITC | | 145-2C11 | BioLegend | 100306 | AB_312671 |  |
| CD4 – Brilliant Violet 605 | | GK1.5 | BioLegend | 100451 | AB_2564591 |  |
| CD8a – Brilliant Violet 650 | | 53-6.7 | BioLegend | 100741 | AB_11124344 |  |
| Foxp3 – PE | | FJK-16s | eBioscience | 12-5773-82 | AB_465936 |  |
| RORγt – Brilliant Violet 421 | | Q31-378 | BD Biosciences | 562894 | AB_2687545 |  |
| CD3e – PE-Cy7 | | 145-2C11 | BD Biosciences | 552774 | AB_394460 |  |
| γδTCR – FITC | | EbioGL3 | eBioscience | 11-5711-82 | AB_465238 |  |
| CD3 – Alexa Fluor 488 | | 17A2 | BioLegend | 100210 | AB_389301 |  |
| TNF-alpha – PE-Cy7 | | TN3-19.12 | eBioscience | 25-7423-82 | AB_494228 |  |
| IL-17A – Brillian Violet 650 | | TC11-18H10.1 | BioLegend | 506927 | AB_11126144 |  |
| CD122 – PE | | 5H4 | BioLegend | 105906 | AB_2125736 |  |
| CD44 – Alexa Fluor 700 | | IM7 | BioLegend | 103026 | AB_493713 |  |
| CD49b – eFluor 450 | | DX5 | eBioscience | 48-5971-82 | AB_10671541 |  |
| CD62L – PerCP-Cy5.5 | | MEL-14 | eBioscience | 45-0621-82 | AB_996667 |  |
| CD69 – Brilliant Violet 510 | | H1.2F3 | BD Biosciences | 563030 | AB_2737963 |  |
| CD3 – eFluor 450 | | | 17A2 | eBioscience | 48-0032-82 | AB_1272193 |
| CD11b – Alexa Fluor 700 | | | M1/70 | eBioscience | 56-0112-82 | AB_657585 |
| CD11c – Brilliant Violet 711 | | | N418 | BioLegend | 117349 | AB_2563905 |
| CD45 – Alexa Fluor 700 | | | 30-F11 | BioLegend | 103128 | AB_493715 |
| CD45R/B220 – Brilliant Violet 510 | | | RA3-6B2 | BioLegend | 103247 | AB_2561394 |
| Ly6G – FITC | | | 1A8 | BioLegend | 127606 | AB_1236494 |
| Ly6C – Brilliant Violet 605 | | | HK1.4 | BioLegend | 128036 | AB_2562353 |
| I-A/I-E – PerCP-Cy5.5 | | | M5/114.15.2 | BioLegend | 107626 | AB_2191071 |
|  | |  |  |  |  |  |


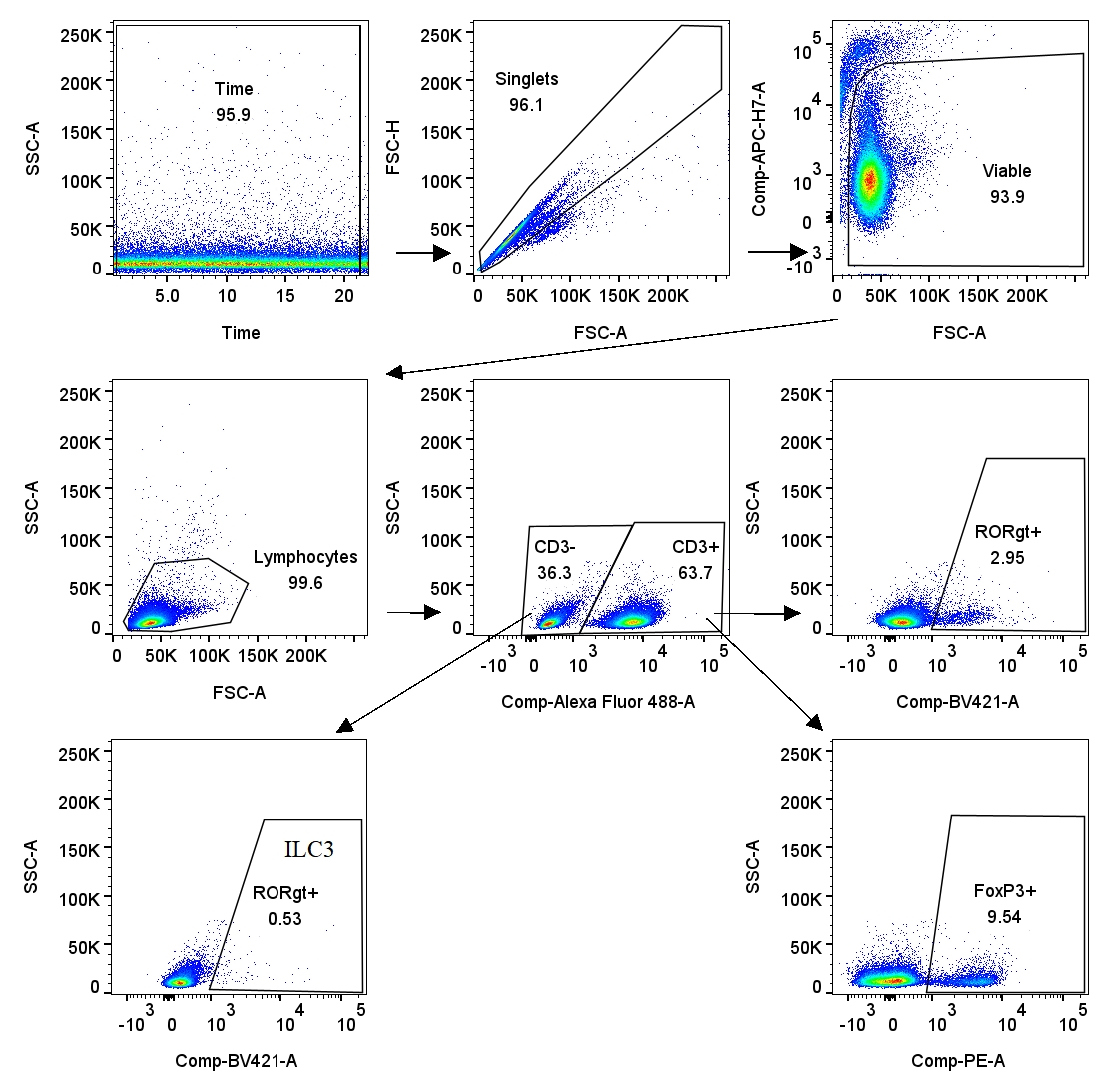


**Supplementary Figure 1.** Gating strategy for Treg cells (CD3^+^Foxp3^+^), Th17 cells (CD3^+^RORγt^+^), and ILC3 (CD3^-^ RORγt^+^) using inguinal lymph nodes of EAE C57BL/6 mouse.

**
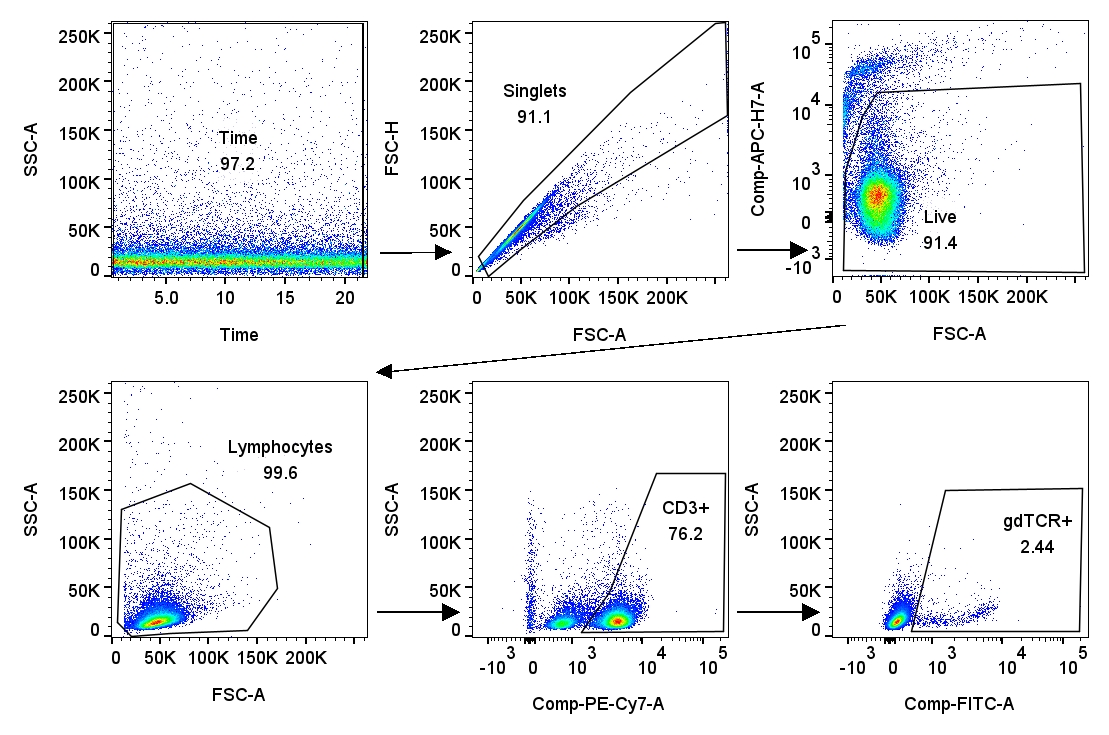
**

**Supplementary Figure 2**. Gating strategy for CD3^+^γδTCR^+^ using inguinal lymph nodes of EAE C57BL/6 mouse.


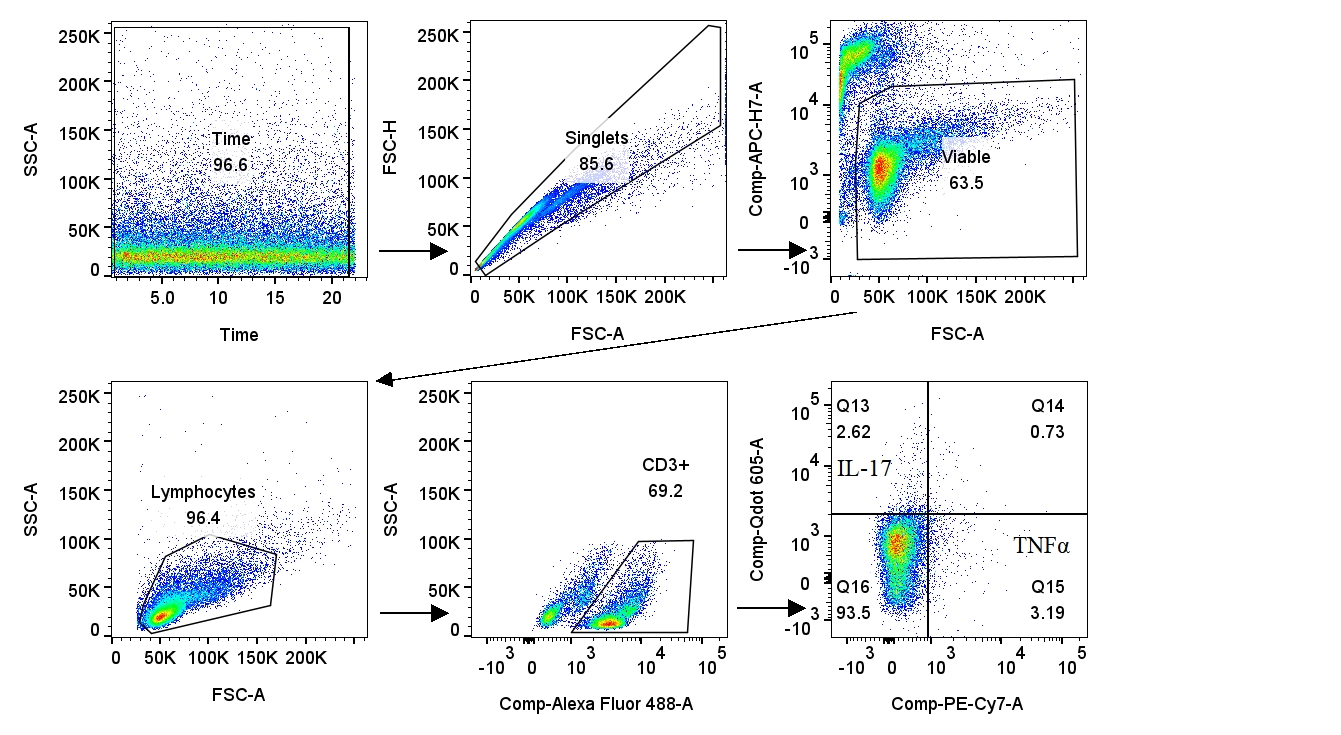


**Supplementary Figure 3**. Gating strategy for CD3^+^TNFα^+^IL17^-^ cells using inguinal lymph nodes of EAE C57BL/6 mouse.

**
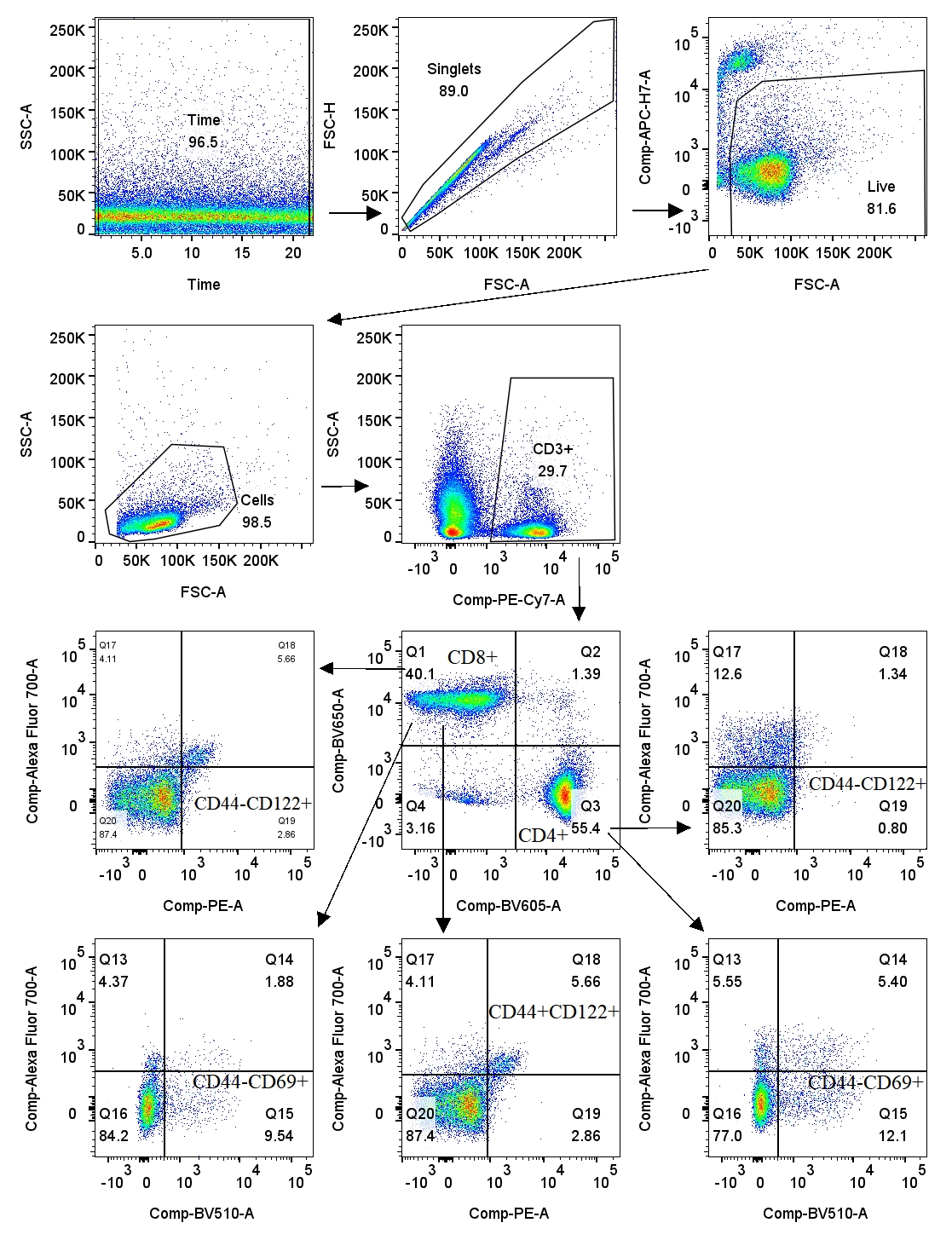
**

**Supplementary Figure 4.** Gating strategy for CD3^+^CD8^+^CD44^+^CD122^+^ cells and for CD3^+^CD4^+^ CD44^-^CD69^+^, CD3^+^CD8^+^ CD44^-^CD69^+,^ CD3^+^CD4^+^CD44^-^CD122^+^, CD3^+^CD8^+^CD44^-^CD122^+^ cells using inguinal lymph nodes of EAE C57BL/6 mouse.


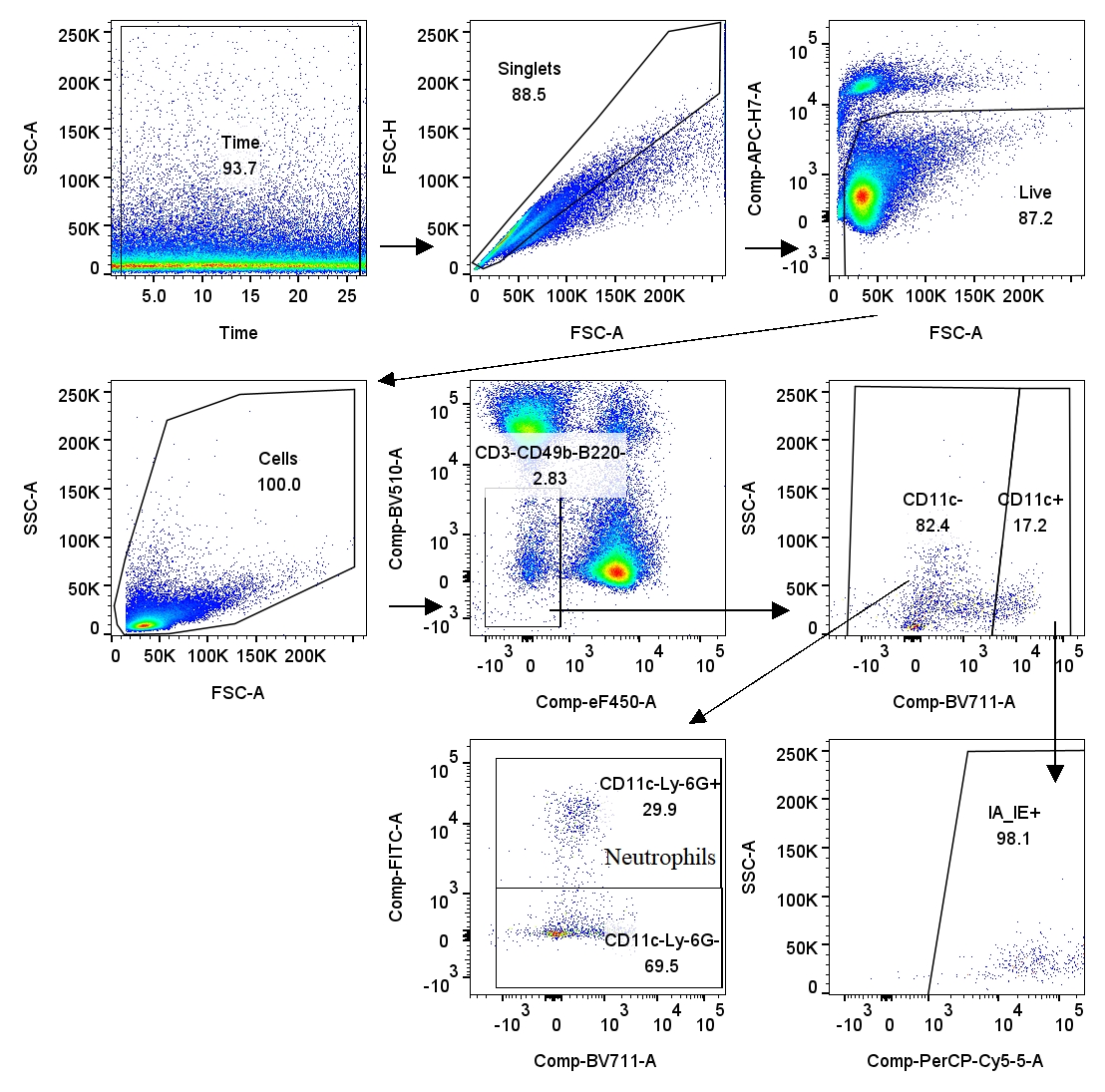


**Supplementary Figure 5.** Gating strategy for neutrophils CD11c^-^Ly6G^+^, dendritic cells CD11c^+^ and CD11c^+^I-A/I-E^+^ using inguinal lymph nodes of EAE C57BL/6 mouse.

**Supplementary Table 2.** PCR primers used for gene expression analysis by quantitative RT-PCR.

| **Name** | **Sequence (5´- 3´)** | **Target** | **References** |
| --- | --- | --- | --- |
| **mouse genes** | | | |
| ***calprotectin*** | CAAGGAAATCACCATGCCCTCTA | *calprotectin* | Origene |
|  | ACCATCGCAAGGAACTCCTCGA |  |  |
| ***Ifng*** | CAGCAACAGCAAGGCGAAAAAGG | *interferon gamma* | Origene |
|  | TTTCCGCTTCCTGAGGCTGGAT |  |  |
| ***Il1b*** | TGGACCTTCCAGGATGAGGACA | *interleukin 1 beta* | Origene |
|  | GTTCATCTCGGAGCCTGTAGTG |  |  |
| ***inos*** | GAGACAGGGAAGTCTGAAGCAC | inducible nitric oxide synthase | Origene |
|  | CCAGCAGTAGTTGCTCCTCTTC |  |  |
| ***Il7*** | CAGACTACCTCAACCGTTCCAC | *interleukin 17* | Origene |
|  | TCCAGCTTTCCCTCCGCATTGA |  |  |
| ***Tnfa*** | GGTGCCTATGTCTCAGCCTCTT | *tumor necrosis factor alpha* | Origene |
|  | GCCATAGAACTGATGAGAGGGAG |  |  |
| ***FoxP3*** | CCTGGTTGTGAGAAGGTCTTCG | *forkhead box P3* | Origene |
|  | TGCTCCAGAGACTGCACCACTT |  |  |
| ***Hprt*** | GGTTAAGCAGTACAGCCCCA | *hypoxanthine guanine phosphoribosyl transferase* | this study |
|  | GGCCTGTATCCAACACTTCG |  |  |
| ***Pgk1*** | CCAAAGGATCAAGGCTGCTG | *phosphoglycerate kinase 1* | this study |
|  | TGCCCAGCAGAGATTTGAGT |  |  |
| ***Tlr4*** | TTCTTCTCCTGCCTGACACC | *Toll-like receptor 4* | (1) |
|  | CTTTGCTGAGTTTCTGATCCAT |  |  |
| ***Muc2*** | CAAACCTGTGCGTGTTCCTG | *mucin 2* | (2) |
|  | GAGCCCATCGAAGGTGACAA |  |  |
| ***Reg3b*** | CTGCCTTAGACCGTGCTTTC | *regenerating islet-derived protein 3-beta* | (3) |
|  | CCCTTGTCCATGATGCTCTT |  |  |
| ***Reg3g*** | TTCCTGTCCTCCATGATCAAAA | *regenerating islet-derived protein 3-gamma* | (4) |
|  | CATCCACCTCTGTTGGGTTCA |  |  |
| ***Il22*** | CAACTTCCAGCAGCCATACA | *interleukin 22* | (5) |
|  | GTTGAGCACCTGCTTCATCA |  |  |
| ***Camp*** | CTTCAACCAGCAGTCCCTAGAC | *cathelicidin antimicrobial peptide* | Origene |
|  | GCCACATACAGTCTCCTTCACTC |  |  |
| **bacteria** | | | |
| **all bacteria** | CGGCAACGAGCGCAACCC | *16S rRNA gene* | (6) |
|  | CCATTGTAGCACGTGTGTAGCC |  |  |
| ***Lactobacillus spp.*** | TGGAAACAGRTGCTAATACCG | *16S rRNA gene of Lactobacillus spp.* | (7) |
|  | GTCCATTGTGGAAGATTCCC |  |  |
| ***P. distasonis*** | GTCGGACTAATACCGCATGAA | *16S rRNA gene of P. distasonis* | (8) |
|  | TTACGATCATAGAACCTTCAT |  |  |
| ***Anaerostipes spp.*** | ACGCGAAGAACCTTACCTGG | *16S rRNA gene of Anaerostipes spp.* | this study |
|  | CCCAACATCTCACGACACGA |  |  |
| **Lachnospiracea/Ruminococaceae** | CGGTACCTGACTAAGAAGC | *16S rRNA gene of Lachnospiracea/Ruminococcaceae* | (9) |
|  | AGTTTYATTCTTGCGAACG |  |  |
| ***Prevotela spp.*** | GAGAGGAAGGTCCCCCAC | *16S rRNA gene of Prevotella spp.* | this study |
|  | CGCTACTTGGCTGGTTCAG |  |  |

**
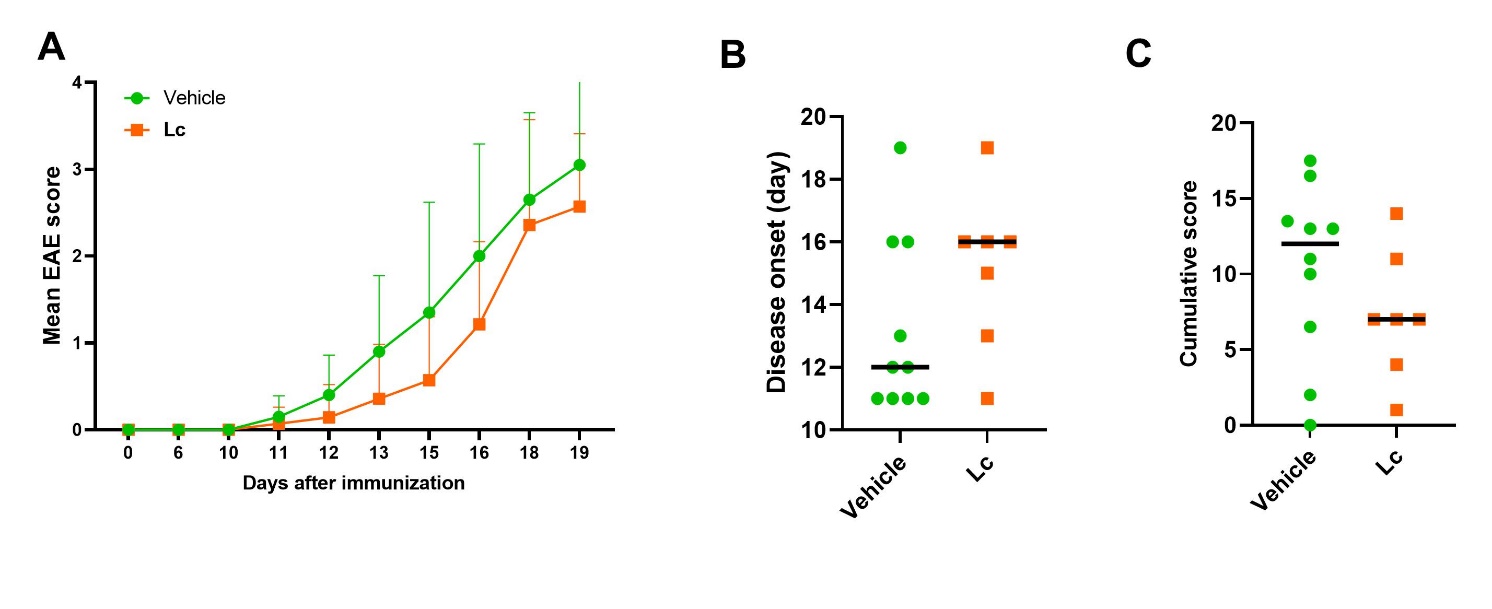
**

**Supplementary Figure 6**. ***Lysate of Lacticaseibacillus casei DN-114 001 (Lc) did not delay the onset and decreas the severity of EAE.***  *(****A****) Clinical disease scores. (****B)*** *Disease onset was defined as the first day a mouse displayed any symptoms. (****C****) Cumulative disease index (CDI; measure of disease severity) depicts the sum of paralysis scores over time for each mouse in all groups. Incidence and cumulative disease scores were tracked through day 19. The graphs show the results of one representative experiment (n = 7–10 mice per group). Statistical significance was determined by unpaired Mann-Whitney t-test. Lc-lysate of Lacticaseibacillus casei DN-114 001*


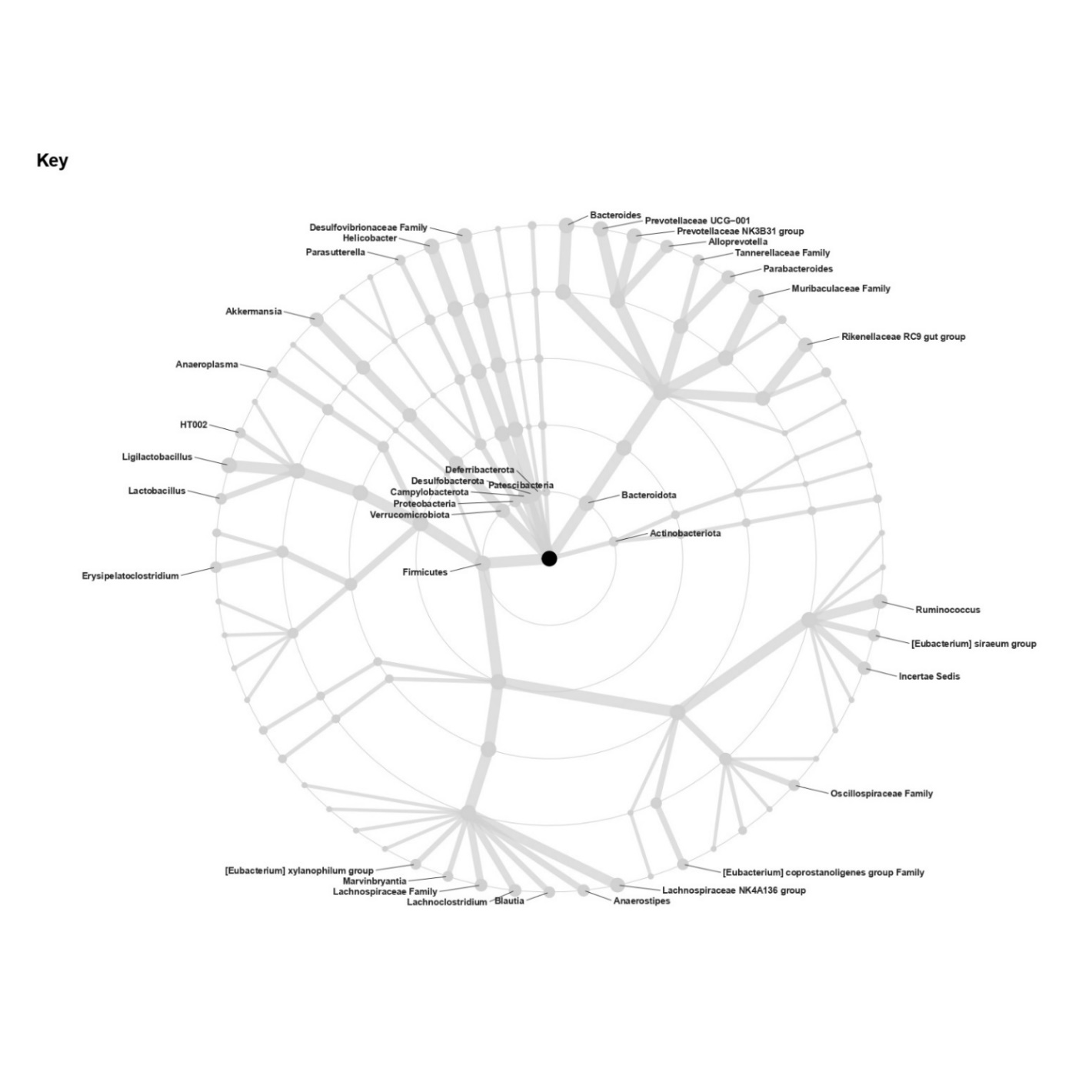


***Supplementary Figure 7. The key for the taxonomical tree for a dedicated time point (day 46 = termination).*** *This key refers to Figure 2E where the statistical differences in abundances of genera between the groups -placebo and Pd group at day 46 experiment termination was performed using the TSS log2 linear regression.*

***
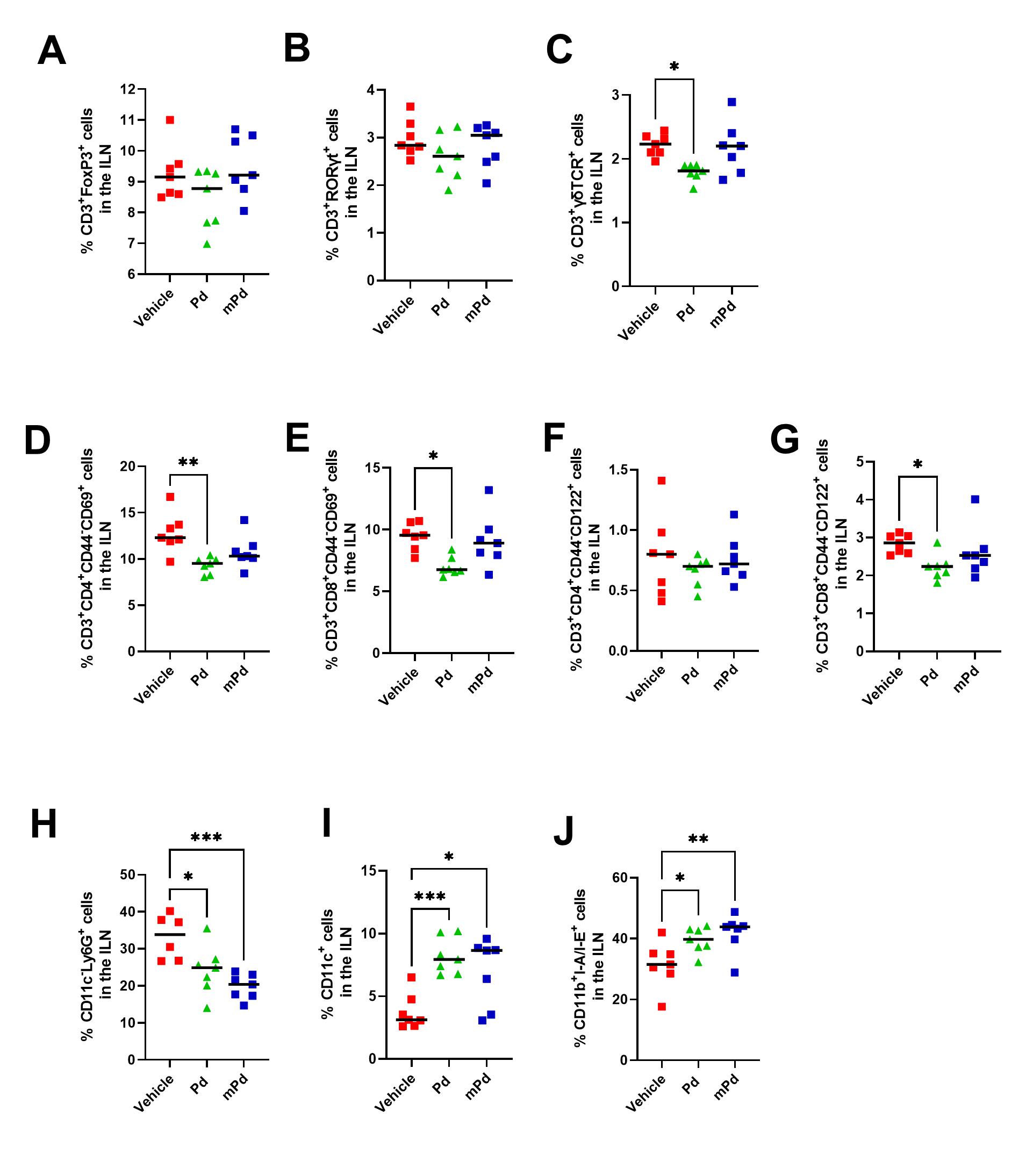
***

***Supplementary Figure 8****.* ***Both*** ***Pd lysate (Pd) or its membranous fraction (mPd) shift T cell populations and phenotype, and alter innate immune cells subsets in distant inguinal lymph nodes (iLN).*** *FACS analysis of inguinal lymph node T cell staining for T reg cells (CD3^+^Foxp3^+^)* ***(A****), CD3^-^RORγt^+^* ***(B****), CD3^+^γδTCR^+^(****C)****, for CD3^+^CD4^+^ CD44^-^CD69^+^ (****D****)CD3^+^CD8^+^ CD44^-^CD69^+^* ***(E****), CD3^+^CD4^+^CD44^-^CD122^+^* ***(F****), CD3^+^CD8^+^CD44^-^CD122^+^ (****G****) cells, and for neutrophils (CD11c^-^Ly6G^+^) (****H****) and dendritic cells (CD11c^+^ and CD11c^+^I-A/I-E^+^) (****I,J****)* ***C****). The data are representative of one out of three independent experiments (n = 5-7 mice per group). Statistical significance was determined by unpaired Student t test *p < 0.05; **p < 0.01, ***p < 0.001.*

**
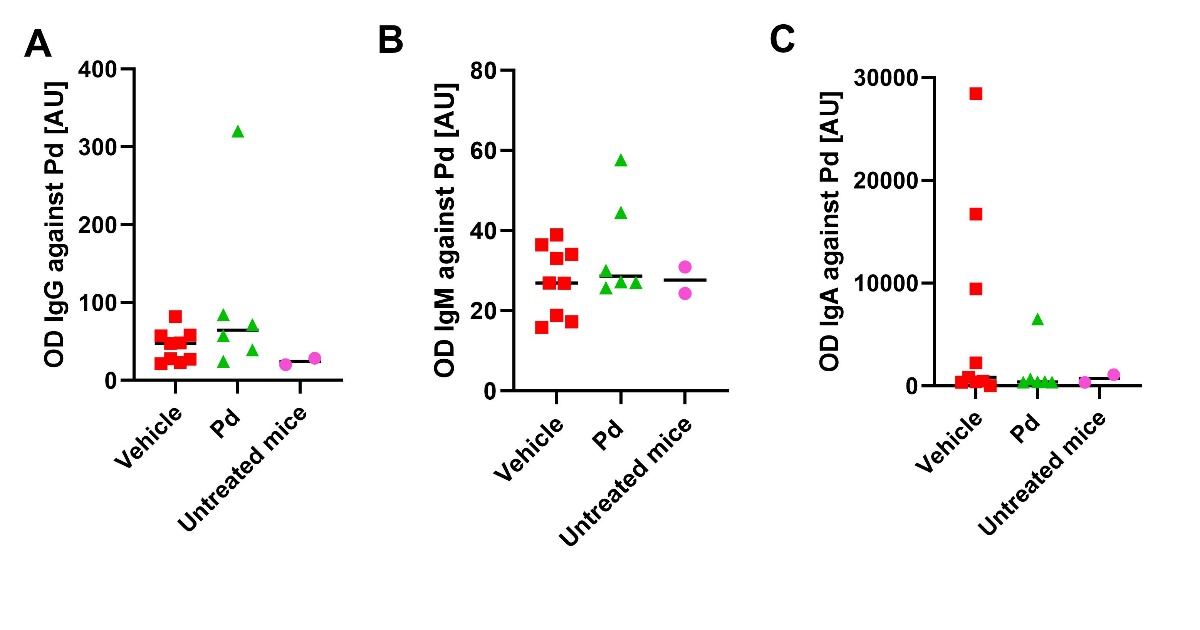
**

***Supplementary Figure 9. Pd lysate does not change the levels of IgG, IgM, IgA antibodies in serum compared to Vehicle group****. Serum titres of anti- IgG (****A****), IgM (****B****), IgA (****C****) P. distasonis antibodies were not changed in mice treated orally with Pd compared to PBS-treated mice and untreated controls. Statistical significance was determined by ANOVA. AU- arbitrary units*

**References**

1. Liu T, Matsuguchi T, Tsuboi N, Yajima T, Yoshikai Y. Differences in expression of toll-like receptors and their reactivities in dendritic cells in BALB/c and C57BL/6 mice. Infect Immun. 2002;70(12):6638-45.

2. Hoebler C, Gaudier E, De Coppet P, Rival M, Cherbut C. MUC genes are differently expressed during onset and maintenance of inflammation in dextran sodium sulfate-treated mice. Dig Dis Sci. 2006;51(2):381-9.

3. Jacobson A, Lam L, Rajendram M, Tamburini F, Honeycutt J, Pham T, et al. A Gut Commensal-Produced Metabolite Mediates Colonization Resistance to Salmonella Infection. Cell Host Microbe. 2018;24(2):296-307 e7.

4. Syder AJ, Oh JD, Guruge JL, O'Donnell D, Karlsson M, Mills JC, et al. The impact of parietal cells on Helicobacter pylori tropism and host pathology: an analysis using gnotobiotic normal and transgenic mice. Proc Natl Acad Sci U S A. 2003;100(6):3467-72.

5. Molinero LL, Cubre A, Mora-Solano C, Wang Y, Alegre ML. T cell receptor/CARMA1/NF-kappaB signaling controls T-helper (Th) 17 differentiation. Proc Natl Acad Sci U S A. 2012;109(45):18529-34.

6. Finotti A, Gasparello J, Lampronti I, Cosenza LC, Maconi G, Matarese V, et al. PCR detection of segmented filamentous bacteria in the terminal ileum of patients with ulcerative colitis. BMJ Open Gastroenterol. 2017;4(1):e000172.

7. Zhang R, Daroczy K, Xiao B, Yu L, Chen R, Liao Q. Qualitative and semiquantitative analysis of Lactobacillus species in the vaginas of healthy fertile and postmenopausal Chinese women. J Med Microbiol. 2012;61(Pt 5):729-39.

8. Wang RF, Cao WW, Cerniglia CE. PCR detection and quantitation of predominant anaerobic bacteria in human and animal fecal samples. Appl Environ Microbiol. 1996;62(4):1242-7.

9. Nava GM, Friedrichsen HJ, Stappenbeck TS. Spatial organization of intestinal microbiota in the mouse ascending colon. ISME J. 2011;5(4):627-38.
